# Computational Design and Glycoengineering of Interferon‐Lambda for Nasal Prophylaxis Against Respiratory Viruses

**DOI:** 10.1002/advs.202506764

**Published:** 2025-11-20

**Authors:** Jeongwon Yun, Seungju Yang, Jae Hyuk Kwon, Luiz Felipe Vecchietti, Ji Hyun Choi, Mi‐ra Choi, Keun Bon Ku, Hyun‐Joo Ro, Kyun‐Do Kim, Meeyoung Cha, Hyun Jung Chung, Ji Eun Oh, Ho Min Kim

**Affiliations:** ^1^ Department of Biological Sciences Korea Advanced Institute of Science and Technology (KAIST) Daejeon 34141 Republic of Korea; ^2^ Graduate School of Medical Science and Engineering Korea Advanced Institute of Science and Technology (KAIST) Daejeon 34051 Republic of Korea; ^3^ Max Plank Institute for Security and Privacy (MPI‐SP) 44799 Bochum Germany; ^4^ School of Computing Korea Advanced Institute of Science and Technology (KAIST) Daejeon 34141 Republic of Korea; ^5^ Center for Infectious Disease Vaccine and Diagnosis Innovation (CEVI) Korea Research Institute of Chemical Technology Daejeon 34114 Republic of Korea; ^6^ Center for Biomolecular and Cellular Structure Institute for Basic Science (IBS) Daejeon 34126 Republic of Korea; ^7^ InnoCORE AI‐CRED Institute Korea Advanced Institute of Science and Technology (KAIST) Daejeon 34141 Republic of Korea

**Keywords:** AI‐based protein design, antiviral prophylaxis, Glyco‐engineering, interferon‐lambda, intranasal biologics

## Abstract

Interferon‐λ (IFN‐λ), a type III interferon that selectively targets epithelial cells, holds strong potential as an intranasal antiviral due to its ability to suppress respiratory virus replication without inducing systemic inflammation. However, clinical translation of human IFN‐λ3 (hIFN‐λ3) is hindered by limited thermostability, protease susceptibility, and rapid mucosal clearance. In this study, instability‐prone elements in hIFN‐λ3 are eliminated through artificial intelligence (AI)‐based backbone remodeling and targeted surface hydrophobic patch engineering. A protease‐sensitive loop is replaced with a *de novo* α‐helix, which shields neighboring hydrophobic patches and forms a new hydrophobic core, yielding an engineered variant (hIFN‐λ3‐DE1) with enhanced thermostability (Tm > 90 °C), protease resistance, and preserved antiviral activity and structural integrity even after extended heat stress (two weeks at 50 °C). Further glyco‐engineering introduces an N‐linked glycan at a site distant from receptor‐binding interfaces, improving solubility, production yield, and diffusion through synthetic nasal mucus. Intranasal administration of the resulting variant (G‐hIFN‐λ3‐DE1) enables effective mucosal penetration and provides a more rapid onset of in vivo prophylactic protection against influenza A virus. These findings highlight a robust and versatile strategy that combines AI‐driven structural design with glyco‐engineering to develop scalable, bioavailable, and functionally enhanced nasal biologics for respiratory virus prophylaxis.

## Graphical abstract

This study presents an engineered human interferon‐lambda (hIFN‐λ) as an intranasal prophylactic against respiratory viruses. By combining AI‐guided backbone redesign and glycoengineering, the authors developed a thermostable, protease‐resistant, and scalable variant with improved mucosal penetration. This engineered hIFN‐λ provides robust antiviral effects in both cell and animal models, demonstrating a promising strategy for next‐generation intranasal prophylactic biologics against respiratory viruses.

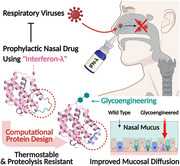

## 1 Introduction

**Figure 1:**
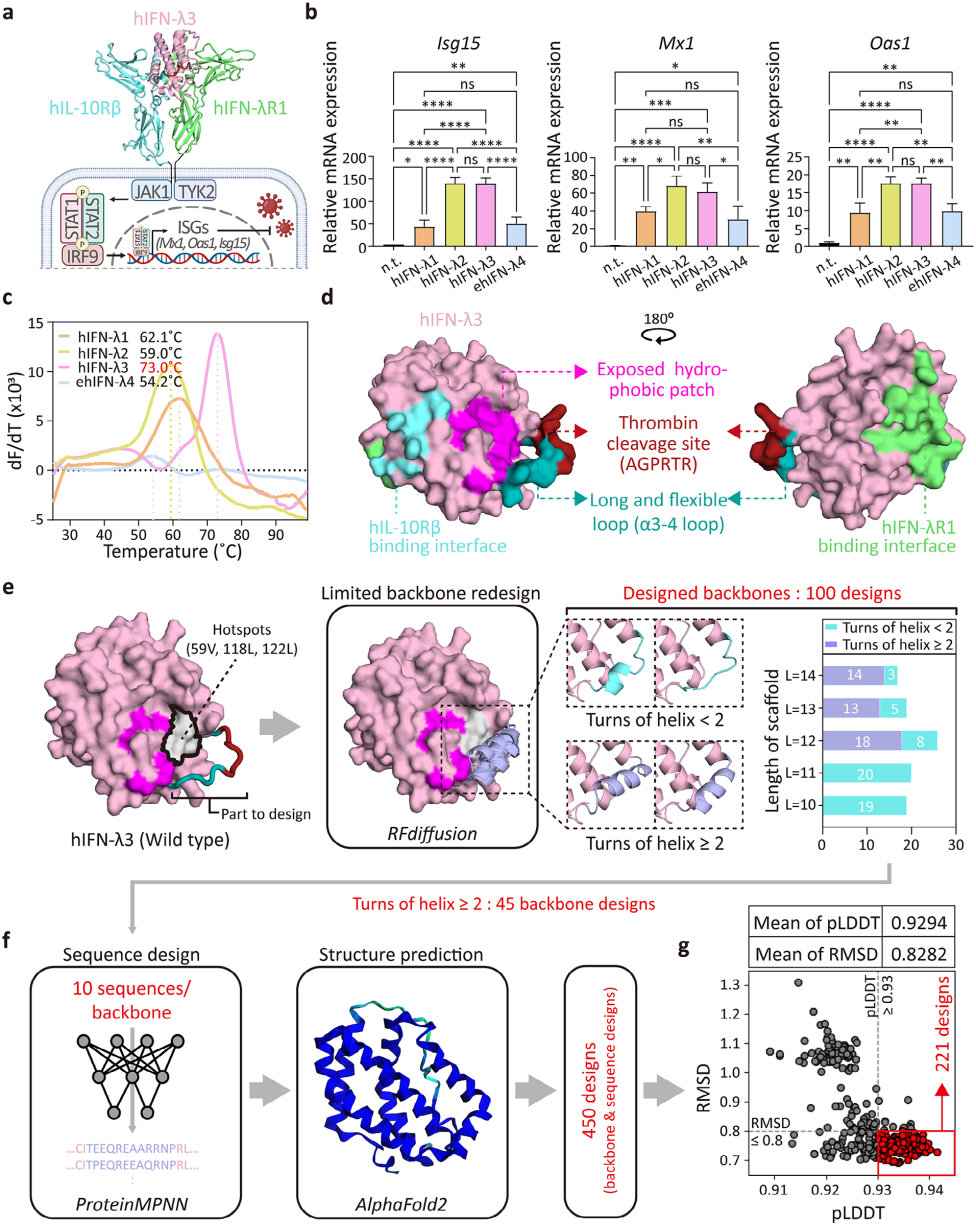
Structure analysis and computational design strategy for thermostable and proteolysis‐resistant hIFN‐λ3. a) The 3D structure of the hIFN‐λ3/hIFN‐λR1/hIL‐10Rβ (PDB code: 5T5W)^[^ ^50^ ^]^ complex and its downstream signaling pathway. b) Induction of representative ISG expression (*Isg15, Mx1, and Oas1*) in HNEpCs after 12‐h treatment with recombinant hIFN‐λs WT, analyzed by RT‐qPCR (*n* = 3). mRNA levels were calculated relative to non‐treated controls and normalized to human *18s rRNA* expression. c) First‐derivative plots (dF/dT) of thermal shift assays for calculating melting temperatures (Tm). 12.5 µg of each hIFN‐λ was mixed with 2.5 µL of diluted Protein Thermal Shift Dye. Tm values, corresponding to the peak dF/dT temperature, are indicated. d) Surface representation of hIFN‐λ3 (PDB code: 3HHC).^[^ ^49^ ^]^ The structure of the α3‐4 loop, which is missing in the crystal structure, is predicted by AlphaFold2 (AF2). The binding sites of hIFN‐λ3 for IL‐10Rβ and IFN‐λR1 are highlighted in cyan and lime, respectively. The exposed hydrophobic patch (magenta), thrombin cleavage site (red), and flexible α3–4 loop (dark green) are shown. e) Targeted backbone redesign using RFdiffusion. The designated hotspot residues (59 V, 118L, and 122L) on the exposed hydrophobic patch for RFdiffusion are highlighted in light gray. The resulting 100 designed backbones are categorized by scaffold length and the number of α‐helix turns at the redesigned backbone. f) Sequence design of 45 redesigned backbones (10 sequences/1 redesigned backbone) using ProteinMPNN, followed by structure prediction with AlphaFold2 (AF2). g) Scatter plot showing AlphaFold2‐predicted confidence (pLDDT) versus RMSD (AF2 prediction compared to the generated backbone) for 450 designed hIFN‐λ3 variants. Dashed lines indicate selection thresholds (pLDDT ≥ 93, RMSD ≤ 0.8), with 221 selected designs in the lower‐right quadrant. All data represent mean ± SD from independent experiments. Statistical analysis was performed using one‐way ANOVA followed by Tukey's multiple comparisons test (0.01<*P<0.1, 0.001<**P<0.01, 0.0001<***P< 0.001, ****P<0.0001 versus control; ns, not significant).

Viral infections pose a persistent and evolving threat to global health, demanding innovative strategies for broadly applicable antiviral therapeutic and prophylactics. Infected viruses are recognized by pattern recognition receptors, including Toll‐like receptors (TLRs) and RIG‐I‐like receptors (RLRs), which trigger the expression of interferons (IFNs)—key cytokines that induce antiviral immunity.^[^ ^1^, ^2^ ^]^ Among the three classes of IFNs (types I, II, and III), type I IFNs (IFN‐α and IFN‐β) are well‐characterized for their systemic antiviral defense mediated via a ubiquitously expressed receptor complex (IFNAR1 and IFNAR2).^[^ ^3^ ^]^ In contrast, type III IFNs (IFN‐λs) signal through a receptor complex (IFN‐λR1 and IL‐10Rβ)^[^ ^4^, ^5^ ^]^ (**Figure** **1**a) predominantly expressed on mucosal surfaces, such as the respiratory and gastrointestinal epithelia, allowing IFN‐λs to induce localized, but potent antiviral responses with minimal systemic inflammation.^[^ ^6^, ^7^, ^8^ ^]^

Emerging evidence underscores the central role of IFN‐λs in respiratory antiviral defense, particularly against high‐burden pathogens such as influenza A virus (IAV), SARS‐CoV‐2, and MERS‐CoV.^[^ ^9^, ^10^, ^11^ ^]^ Clinical studies have demonstrated that elevated levels of IFN‐λ correlate with reduced viral loads, enhanced viral clearance, and improved clinical outcomes in COVID‐19 patients,^[^ ^12^, ^13^, ^14^ ^]^ proposing its frontline role in suppressing viral spread within the respiratory epithelium.^[^ ^15^, ^16^ ^]^ These findings highlight IFN‐λ as a promising candidate for broad‐spectrum antiviral therapeutics targeting the respiratory epithelium.

Nevertheless, recent clinical trials of subcutaneously administered IFN‐λ have reported mixed outcomes, with modest antiviral efficacy and variable patient responses.^[^ ^17^, ^18^, ^19^ ^]^ Such limitations are attributed to inadequate drug levels at the primary sites of viral infection—the nasal and upper respiratory epithelium—due to poor delivery, as well as delayed timing of administration. Many patients were enrolled ≈5 days after symptom onset, with over 40% already seropositive, making early intervention difficult. Earlier or prophylactic administration might therefore have led to better outcomes. Moreover, although higher or more frequent dosing could enhance efficacy, subcutaneous doses above 180 µg are constrained by increasing toxicity, including significant elevations in liver transaminases.

Similarly, the clinical application of type I IFNs, such as IFN‐α and IFN‐β, has also faced significant challenges. During the COVID‐19 pandemic, clinical trials (e.g., WHO Solidarity Trial) reported minimal benefit of IFN‐β1a in hospitalized patients, leading to early discontinuation of these treatment arms.^[^ ^20^ ^]^ Delayed administration of IFN‐α was associated with increased mortality and prolonged recovery, underscoring a narrow therapeutic window.^[^ ^21^ ^]^ In addition, type I IFNs have been implicated in exacerbating hyperinflammation in severe COVID‐19, raising concerns about immunopathological effects.^[^ ^22^ ^]^ These findings underscore the risks of systemic type I IFN therapy, especially in advanced stages of disease, and highlight the translational need for safer, targeted antiviral strategies.

Nasal delivery, a non‐invasive and efficient route to the site of respiratory virus infection and replication, offers the potential for IFN‐λ‐based therapies.^[^ ^23^, ^24^ ^]^ Unlike systemic administration, intranasal delivery enables direct access to epithelial IFN‐λ receptors, ensuring rapid antiviral action while minimizing systemic exposure. Recent studies have highlighted the efficacy of intranasally delivered recombinant IFN‐λ2 in reducing viral loads and mitigating lung damage caused by SARS‐CoV‐2 variants.^[^ ^25^ ^]^ Despite this promise, the development of nasal protein therapeutics encounters significant challenges, including protein instability, degradation by nasal proteases, and rapid mucociliary clearance.^[^ ^26^, ^27^, ^28^ ^]^

In this study, we employed AI‐based protein design and glyco‐engineering to overcome these barriers and enhance the therapeutic fitness of human IFN‐λ. Our engineered IFN‐λ exhibits remarkable stability, with a melting temperature (Tm) exceeding 90 °C and resistance to proteolytic degradation, retaining full biological activity even after two weeks incubation at 50 °C. Additionally, the engineered IFN‐λ demonstrates superior production yields and improved diffusion efficiency across the mucosal barrier. Intranasal administration of this engineered IFN‐λ provided robust protection against IAV infection in mouse models, significantly reducing viral burden and associated pathology. These findings establish a new paradigm for the development of protein‐based nasal therapeutics, offering a scalable, stable, and effective prophylactic solution against respiratory viruses, including emerging variants. Moreover, our work underscores the transformative potential of computational protein design in addressing critical challenges in antiviral drug development, facilitating the way for next‐generation mucosal immunotherapies.

## 2 Results

### 2.1 Comparative Analysis Identifies hIFN‐λ3 as an Optimal Antiviral Candidate for Enhancing Protein Fitness

Humans possess four functional IFN‐λ genes (IFN‐λ1 to IFN‐λ4), each encoding a cytokine with varying antiviral potency and biophysical characteristics. To identify the most suitable candidate for developing prophylactic intranasal biologics against respiratory viruses, we conducted a comparative analysis of all four human IFN‐λ proteins. Recombinant hIFN‐λ proteins (human IFN‐λ1,2,3, and 4) were produced in mammalian cells and purified via affinity and size‐exclusion chromatography (SEC) (Figure S1a, Supporting Information). For hIFN‐λ4, we expressed a glycoengineered version (eIFN‐λ4) as previously described,^[^ ^29^ ^]^ since the wild‐type hIFN‐λ4 is rarely expressed. SDS‐PAGE and SEC analysis revealed molecular weights ranging from 20 to 30 kDa, with hIFN‐λ1 and eIFN‐λ4 being ≈5–10 kDa larger than hIFN‐λ2 and hIFN‐λ3 due to differential glycosylation (Figure S1a, Supporting Information).

To select the optimal hIFN‐λ subtype for nasal delivery, we prioritized in vitro functional activity and thermal stability. Previous studies have shown that IFN‐λ3 has the lowest binding affinity for IFN‐λR1, compared to IFN‐λ1 and IFN‐λ2 (IFN‐λ1 K_D_ = 15.7 nm; IFN‐λ2 K_D_ = 19.3 nm; IFN‐λ3 K_D_ = 64.7 nm), yet exhibits the highest antiviral potency in vitro.^[^ ^30^, ^31^ ^]^ This indicates that binding affinity alone does not predict biological efficacy. We therefore focused on STAT1 phosphorylation and ISG induction in primary human nasal epithelial cells as direct measures of functional antiviral activity. Thermal stability, quantified by melting temperature, was also assessed to ensure protein integrity during storage and after intranasal delivery. Parameters such as systemic safety and half‐life were not considered in this initial subtype selection, since our aim was to achieve effective local delivery with minimal systemic exposure at the lowest effective dose.

To assess functional activity, we treated primary human nasal epithelial cells (HNEpCs) with each recombinant cytokine and measured STAT1 phosphorylation and induction of key interferon‐stimulated genes (ISGs: *Isg15, Mx1, and Oas1*). All four IFN‐λ proteins activated STAT1 phosphorylation to a similar extent; however, hIFN‐λ2 and hIFN‐λ3 induced stronger ISG responses, indicating superior antiviral signaling (Figure 1b; Figure S1b, Supporting Information). In parallel, we evaluated thermal stability by measuring melting temperature (Tm) with Thermal shift assays. Among the four proteins, hIFN‐λ3 exhibited the highest thermal stability, with a Tm of 73.0 °C, which is substantially higher than hIFN‐λ1 (62.1 °C), hIFN‐λ2 (59.0 °C), and eIFN‐λ4 (54.2 °C) (Figure 1c; Figure S1c, Supporting Information). Based on its robust ISG induction and superior thermal stability, hIFN‐λ3 was selected as the lead antiviral candidate for structure‐guided computational design aimed at further enhancing protein fitness for nasal delivery.

### 2.2 Structure‐Guided Computational Design of Thermostable and Proteolysis‐Resistant hIFN‐λ3

To elucidate structural features relevant to stability and guide rational design, we analyzed the crystal and AlphaFold2 (AF2)^[^ ^32^ ^]^‐predicted structures of human IFN‐λ1, 2, 3, and 4. These proteins share a conserved fold consisting of five α‐helices and two 3_10_ helices connected by flexible loops. The N‐terminal helix (α1) and the C‐terminal helix (α5 of hIFN‐λ1, 2, and 3 and α4 of hIFNL4) interact with IFN‐λR1, whereas the α1‐α3 helices interact with IL‐10Rβ (Figures S2 and S3, Supporting Information). Of note, a flexible loop connecting α3 and α4 helices (the α3‐4 loop) contains a thrombin cleavage sequence.^[^ ^33^ ^]^ Adjacent to this flexible α3‐4 loop, several conserved hydrophobic residues (eg., V59, A63, A66, L97, A102, I104, L118, and L122 in hIFN‐λ3) on helices α2–α4 form a solvent‐exposed hydrophobic patch (Figure 1d; Figures S2 and S3, Supporting Information), which does not participate in receptor binding. Solvent‐exposed hydrophobic patches are known to negatively affect protein thermodynamic stability.^[^ ^34^, ^35^, ^36^, ^37^ ^]^ Therefore, we hypothesized that masking this patch through loop redesign could improve overall stability and proteolytic resistance.

To address this, we sought to replace the flexible α3‐4 loop of hIFN‐λ3 (105Q–116R) with a stable α‐helix that eliminates the thrombin cleavage sequence and buries the hydrophobic patch through internal core interactions. Using RFdiffusion,^[^ ^38^ ^]^ we performed backbone redesigns at this loop position with lengths ranging from 10 to 14 residues. Three hydrophobic residues at the center of the patch (V59, L118, L122) were designated as design hotspots to guide packing interactions for shielding the hydrophobic patch. A total of 100 backbone models at the α3‐4 loop position were generated across four diffusion time steps (T = 50, 100, 150, and 200) (Figure 1e). Secondary structure analysis with the DSSP algorithm^[^ ^39^, ^40^ ^]^ revealed that loops of 12–14 residues were most effective at forming stable α‐helices. From this subset, 45 backbones containing rigid α‐helices with ≥8 helical residues were selected for further sequence design using ProteinMPNN,^[^ ^41^ ^]^ generating 10 unique sequences per backbone while keeping the remaining hIFN‐λ3 sequence fixed. The structures of 450 designs were predicted by AF2^[^ ^32^ ^]^ (Figure 1f), resulting in an average predicted local distance difference test (pLDDT) value of 0.9294 and root mean square deviation (RMSD) of 0.8282. To ensure high confidence and structural fidelity, we applied these average values as filtering metrics (i.e., pLDDT ≥0.93 and RMSD ≤0.8) for further analysis, narrowing the pool to 221 designs (Figure 1g).

**Figure 2:**
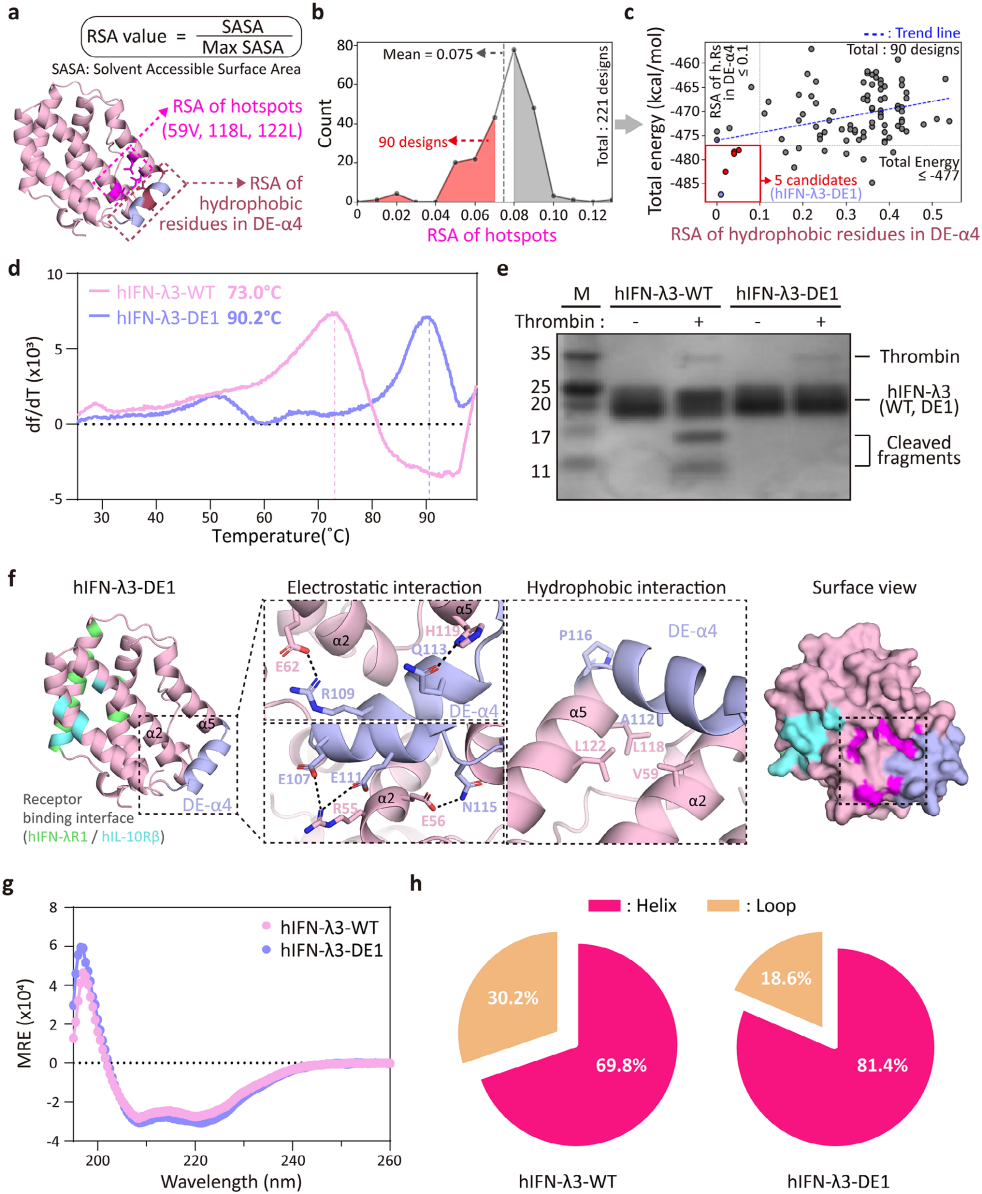
Selection strategy for hIFN‐λ3 designs, followed by evaluation of their increased thermal stability, proteolytic resistance, and structural characterization. a) Schematic showing the calculation of relative solvent accessibility (RSA), defined as the ratio of solvent‐accessible surface area (SASA) to the maximum possible SASA (Max SASA) at the designated area. The regions used for RSA calculation of hotspot residues (V59, L118, and L122) and hydrophobic residues in DE‐α4 are indicated in magenta and brown, respectively. b) Histogram showing the distribution of RSA values for the hotspot residues among 221 designed variants. The average “RSA of hotspots” was 0.075, 90 designs below this threshold are highlighted in red. c) Scatter plot of values for “RSA of hydrophobic residues in DE‐α4” versus “total energy (kcal/mol)”. The regression line (y = 16.122x – 476.08, R^2^ = 0.1251) is shown. Five designs satisfying both selection criteria (RSA ≤ 0.1 and total energy ≤ –477) are indicated in red. d) First derivative (dF/dT) curves of thermal shift assays for determining melting temperature (Tm). The Tm values of hIFN‐λ3‐WT and hIFN‐λ3‐DE1 determined are indicated at peak positions. e) Thrombin susceptibility assay. hIFN‐λ3‐WT and hIFN‐λ3‐DE1 were incubated with thrombin (1% [v/v] in PBS) at 37 °C for 1 h and analyzed by SDS‐PAGE followed by Coomassie blue staining. f) AF2‐predicted model of hIFN‐λ3‐DE1. Insets highlight stabilizing electrostatic (left) and hydrophobic (middle) interactions between the DE‐α4 helix (purple) and neighboring helices (pink). Surface view shows the buried hydrophobic patch (magenta) shielded by DE‐α4 (purple). The binding sites of hIFN‐λ3‐DE1 for IL‐10Rβ and IFN‐λR1 are highlighted in cyan and lime. g) Circular dichroism (CD) spectra of hIFN‐λ3‐WT and hIFN‐λ3‐DE1 at 25 °C across 195–260 nm. h) Pie chart showing the secondary structure content of hIFN‐λ3‐WT and hIFN‐λ3‐DE1 calculated from CD analysis.

To assess whether the redesigned α‐helix (termed DE‐α4) effectively shielded the hydrophobic patch, we calculated the relative solvent accessibility (RSA) of the hotspot residues^[^ ^42^, ^43^ ^]^ (**Figure** **2**a). Although no strict RSA threshold exists, we reasoned that reducing solvent exposure of hydrophobic regions would improve folding stability. Designs with average RSA < 0.075 across the hotspot residues were prioritized (Figure 2b). Additionally, to prevent unintended surface exposure of new hydrophobic residues introduced by DE‐α4, RSA values of hydrophobic residues in the redesigned helix were evaluated. Lastly, we calculated a total free energy using the Rosetta energy function^[^ ^44^, ^45^ ^]^ after structural refinement through Rosetta relaxation.^[^ ^46^, ^47^ ^]^ Interestingly, there was a weak correlation between RSA of hydrophobic residues on DE‐α4 and total free energy (Figure 2c). Based on these integrated criteria (RSA of hotspots ≤ 0.1 and Rosetta energy ≤ –477), we selected the five most promising candidates (hIFN‐λ3‐DE1 to DE5; Figure 2c; **Table** **1** and Tables S2 and S3, Supporting Information).

**Table 1:** Comparison of filtering metrics for wild‐type and designed hIFN‐λ3 candidates.

hIFN‐λ3 (DE‐#)	RSA of Hotspots	RSA of Hydrophobic Residues in DE‐α4	Total Energy (kcal/mol)	ΔE (kcal/mol)
Wild type	0.23	–	−423.434	0
DE‐1	0.06	0.01	−487.285	−63.851
DE‐2	0.07	0.05	−478.023	−54.589
DE‐3	0.06	0.04	−478.305	−54.871
DE‐4	0.07	0.04	−478.717	−55.823
DE‐5	0.07	0.02	−482.502	−59.068

ΔE = ‘Total Energy‘ of wild type – ‘Total Energy‘ of hIFN‐λ3‐DE.

Synthetic genes encoding five designed hIFN‐λ3s were expressed in a mammalian cell and purified (Figure S4a, Supporting Information). All five designs exhibited improved thermal stability compared to wild‐type hIFN‐λ3 (WT), as assessed by thermal shift assays. The most pronounced enhancement was observed for hIFN‐λ3‐DE1, which showed a melting temperature (Tm) of 90.2 °C—substantially higher than that of hIFN‐λ3‐WT (Tm = 73.0  °C) (Figure 2d; Figure S4b, Supporting Information). This result indicates effective structural stabilization via the DE‐α4 design. Furthermore, thrombin cleavage analysis revealed that hIFN‐λ3‐WT was cleaved into two fragments, whereas hIFN‐λ3‐DE1 remained intact, demonstrating resistance to proteolysis (Figure 2e). This resistance is attributed to the removal of the thrombin cleavage motif in the α3‐4 loop in hIFN‐λ3‐DE1 and its structural replacement with a well‐packed α‐helix. Collectively, these results demonstrate that structure‐guided computational design can substantially improve the stability and protease resistance of hIFN‐λ3. The enhanced biophysical properties of hIFN‐λ3‐DE1 support its suitability for protease‐rich mucosal environments, underscoring its potential as a robust intranasal biologic.

### 2.3 Structural Insights into Designed hIFN‐λ3

To elucidate the molecular basis of the enhanced thermal stability observed in hIFN‐λ3‐DE1, we examined the AF2‐predicted structures of the designed hIFN‐λ3s (Figure 2f; Figure S4c, Supporting Information). The hIFN‐λ3‐DE1 structure revealed newly formed intramolecular interactions between the designed DE‐α4 and adjacent helices (α2 and α5), contributing to structural stabilization (Figure 2f). Notably, a sharp kink at P116 in DE‐α4, along with multiple electrostatic interactions (E62^α2^‐R109^DE‐α4^, R55^α2^‐E107^DE‐α4^ and E111^DE‐α4^, H119^α5^‐Q113^DE‐α4^, and E56^α2^‐N115^DE‐α4^) locked DE‐α4 into a fixed orientation. In addition, A112^DE‐α4^ formed hydrophobic contacts with three hotspot residues (V59, L118, and L122), thereby effectively burying the hydrophobic patch within the protein core.

We next validated the structural formation of the designed helical element in hIFN‐λ3‐DE1 using circular dichroism (CD) spectroscopy. Secondary structure analysis showed a marked increase in helical content—from 69.8% in hIFN‐λ3‐WT to 81.4% in hIFN‐λ3‐DE1 (Figure 2g,h), supporting the successful loop‐to‐helix transformation introduced by the DE‐α4 design of hIFN‐λ3.

Additionally, to assess whether the insertion of DE‐α4 induces any global structural changes, we compared ten AlphaFold3^[^ ^48^ ^]^‐predicted models of hIFN‐λ3‐WT and hIFN‐λ3‐DE1 to the crystal structure of wild‐type hIFN‐λ3 (PDB: 3HHC)^[^ ^49^ ^]^ (Figure S5a, Supporting Information). Local RMSD values for the receptor‐binding interface and the remainder of the protein (excluding the designed region) were similar between variants (1.545 and 1.787 Å for hIFN‐λ3‐DE1; 1.557 Å and 1.927 Å for hIFN‐λ3‐WT), indicating that the DE‐α4 helix does not cause notable structural deviation (Figure S5b, Supporting Information).

We further evaluated potential effects on receptor binding by modeling ternary complexes of each variant with hIFN‐λR1 and hIL‐10Rβ. For each variant, ten predicted complex structures were aligned to the crystal structure of the hIFN‐λ3 receptor complex (PDB: 5T5W)^[^ ^50^ ^]^ (Figure S5c, Supporting Information). The average RMSD values for the ligand were 1.074 Å (WT) and 1.037 Å (DE1), and for the receptors, 1.112 Å (WT) and 1.106 Å (DE1), demonstrating that the designed DE‐α4 helix does not disrupt the receptor‐binding conformation (Figure S5d, Supporting Information). Indeed, binding affinities of wild‐type hIFN‐λ3 and hIFN‐λ3‐DE1 for IFN‐λR1 were comparable (Figure S5e, Supporting Information). Collectively, these results demonstrate that the engineered DE‐α4 helix enhances the thermal stability of hIFN‐λ3 without inducing significant global structural changes or disrupting receptor‐binding affinity and conformation.

### 2.4 Designed hIFN‐λ3 Retained Biological Activity Under Heat Stress and Long‐Term Storage

Next, we examined whether hIFN‐λ3‐DE1 retains its biological activity in human nasal epithelial cells (HNEpCs). STAT1 phosphorylation levels were comparable between hIFN‐λ3‐WT and hIFN‐λ3‐DE1 (Figure S6a, Supporting Information), suggesting that designed DE‐α4 of hIFN‐λ3‐DE1 preserved the structural integrity of critical functional domains, including receptor‐binding interfaces, thereby maintaining full biological activity.

**Figure 3:**
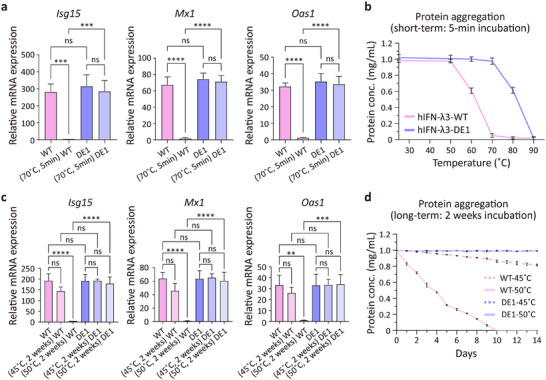
Biological activity and thermal aggregation resistance of hIFN‐λ3‐DE1 under acute and long‐term heat stress. a) Relative mRNA expression of representative ISGs (*Isg15, Mx1, and Oas1*) in HNEpCs following 12‐h treatment with hIFN‐λ3‐WT or hIFN‐λ3‐DE1 (100 ng mL^−1^), with or without short‐term heat stress (70 °C for 5 min). mRNA levels were analyzed by RT‐qPCR (*n* = 3), normalized to *18s rRNA*, and expressed relative to non‐treated controls. b) Short‐term thermal aggregation profiles of hIFN‐λ3‐WT and hIFN‐λ3‐DE1 after 5‐min incubation at the indicated temperatures (25, 50, 60, 70, 80, or 90 °C). Residual soluble protein concentrations were quantified (n = 3). c) Relative ISG expression (*Isg15, Mx1, and Oas1*) in HNEpCs treated with hIFN‐λ3‐WT or hIFN‐λ3‐DE1 (100 ng mL^−1^) after long‐term incubation at 45 or 50 °C for 2 weeks. RT‐qPCR was performed as in (a) (*n* = 3). d) Long‐term thermal aggregation of hIFN‐λ3‐WT and hIFN‐λ3‐DE1 during 2‐week incubation at 45 or 50 °C. Protein solubility was monitored over time (*n* = 3). All data represent mean ± SD from independent experiments. Statistical analysis was performed by one‐way ANOVA followed by Sidak's multiple comparisons test (0.001<**P<0.01, 0.0001<***P < 0.001, ****P<0.0001 vs control and ns is not significant). n.t., non‐treat; WT, hIFN‐λ3‐WT; DE1, hIFN‐λ3‐DE1.

We further assessed resistance to acute heat stress by incubating the proteins at 70 °C for 5 min prior to HNEpC treatment. Notably, hIFN‐λ3‐WT lost all ISG‐inducing activity, whereas hIFN‐λ3‐DE1 retained full activity, equivalent to unheated controls (**Figure** **3**a). Furthermore, aggregation analysis revealed that hIFN‐λ3‐WT aggregated by ≈40% at 60 °C and >90% at 70 °C within 5 min. In contrast, hIFN‐λ3‐DE1 exhibited remarkable resistance to thermal aggregation, maintaining solubility even at 70 °C, with minimal aggregation only at 80 °C (Figure 3b; Figure S6b, Supporting Information).

To evaluate long‐term thermal stability relevant to nasal drug development, we incubated both proteins for 2 weeks at elevated temperatures. Both hIFN‐λ3‐WT and hIFN‐λ3‐DE1 retained ISG‐inducing activity after 2 weeks at 37 °C, comparable to their respective unstressed controls (Figure S6c, Supporting Information). However, hIFN‐λ3‐WT exhibited reduced activity after 2 weeks at 45 °C and lost activity entirely at 50 °C. In contrast, hIFN‐λ3‐DE1 maintained full biological activity even after 2 weeks at both 45 and 50 °C (Figure 3c). Aggregation analysis revealed that hIFN‐λ3‐WT aggregated by ∼20% at 45 °C and reached complete aggregation within 10 days at 50 °C, while hIFN‐λ3‐DE1 remained soluble even after 2 weeks at 45 and 50 °C (Figure 3d). Taken together, these results highlight the superior thermostability of hIFN‐λ3‐DE1, which maintains both structural integrity and biological activity under rigorous storage and heat stress conditions, underscoring its translational potential as a stable and effective intranasal biologic.

### 2.5 Glyco‐Engineering Improves Production and Mucosal Penetration of Designed hIFN‐λ3

Glyco‐engineering has emerged as a powerful strategy to enhance protein therapeutics by improving solubility, folding stability, and bioavailability, while also reducing aggregation and proteolytic susceptibility under physiological conditions.^[^ ^51^, ^52^, ^53^, ^54^ ^]^ PEGylation, a synthetic mimic of glycosylation, has long been utilized to extend protein half‐life and improve diffusion through biological barriers, including the mucus.^[^ ^55^, ^56^, ^57^, ^58^ ^]^ Notably, both PEGylation and glycosylation can create hydrophilic, sterically shielding surfaces that reduce muco‐adhesion and facilitate rapid mucosal penetration.^[^ ^59^, ^60^, ^61^ ^]^ Here, we sought to apply site‐specific glyco‐engineering to our optimized IFN‐λ3 variant (hIFN‐λ3‐DE1) for enhancing its manufacturability and mucosal delivery properties as an intranasal biologic.

**Figure 4:**
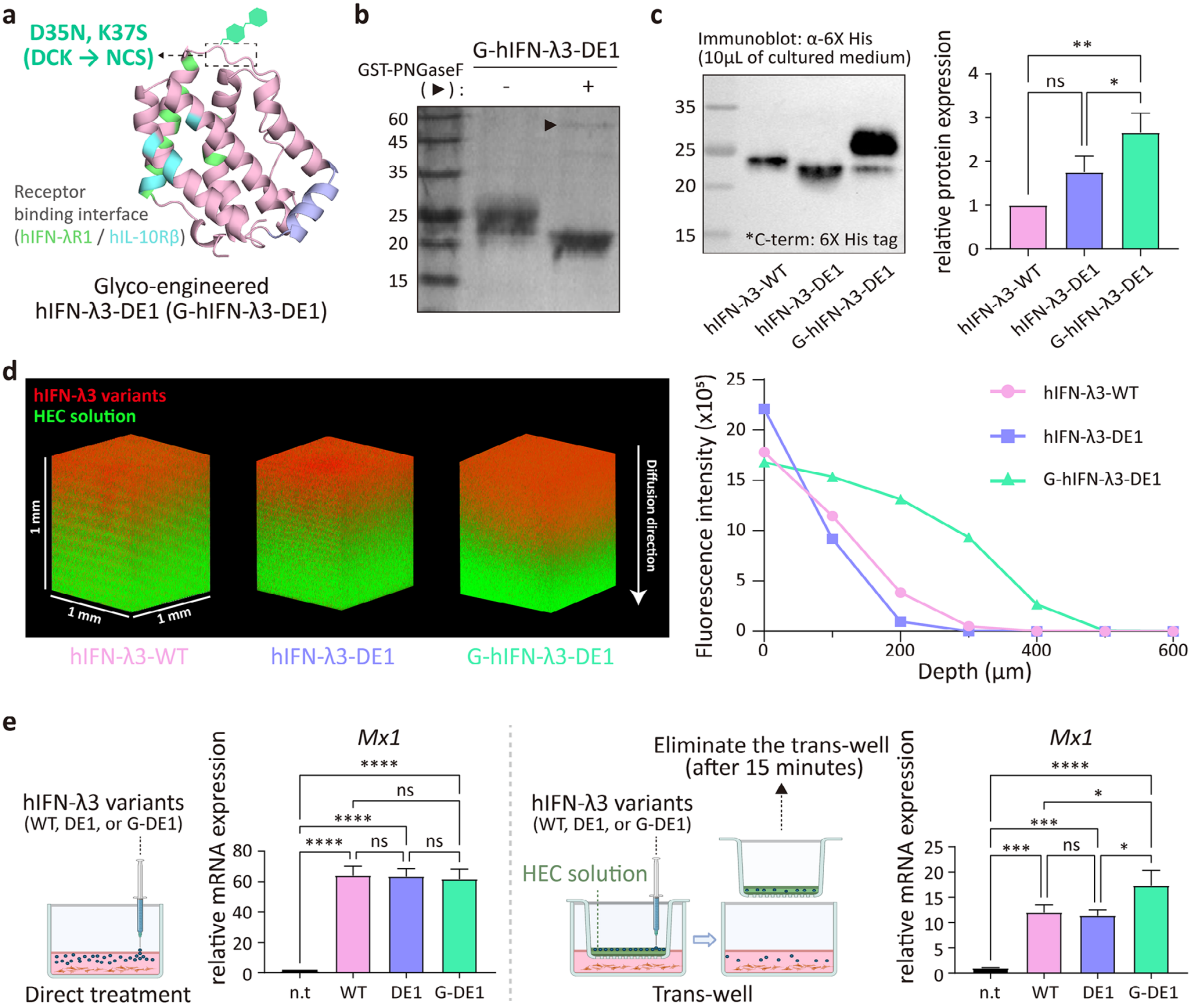
Glyco‐engineering enhances production yield and mucosal diffusion of designed hIFN‐λ3. a) Structure‐guided glyco‐engineering of hIFN‐λ3‐DE1 to generate the glycosylated variant G‐hIFN‐λ3‐DE1. The mutated amino acid residues for N‐glycosylation (D35N/K37S) are indicated. The binding sites of G‐hIFN‐λ3‐DE1 for IL‐10Rβ and IFN‐λR1 are highlighted in cyan and lime. b) SDS‐PAGE analysis of purified G‐hIFN‐λ3‐DE1 before and after PNGaseF‐mediated deglycosylation. c) Expression of hIFN‐λ3 variants (WT, DE1, and G‐DE1) in Expi293F cells. Same volumes of culture supernatants were collected 4 days post‐transfection and analyzed by immunoblotting using an anti‐His antibody (*n* = 3). All hIFN‐λ3 variants contained a C‐terminal 6X‐His tag. d) Confocal imaging of Alexa 647‐labeled hIFN‐λ3 variants (WT, DE1, and G‐DE1) diffusing over 10 min through a 1.2% hydroxyethyl cellulose (HEC) matrix pre‐labeled with BDP‐FL (left). Fluorescence intensity across z‐depth is quantified (right). e) Relative *Mx1* mRNA expression in HNEpCs after 12‐hr incubation with hIFN‐λ3 variants (100 ng mL^−1^, WT, DE1, and G‐DE1). Cells were either treated directly (left) or via a trans‐well diffusion system with 500 µm‐thick HEC layer (right). In the trans‐well setup, protein was applied to the upper insert and removed after 15 min and cells were then incubated for 12 h prior to RT‐qPCR (*n* = 3). All data represent mean ± SD from independent experiments. Statistical significance was determined by one‐way ANOVA followed by Tukey's multiple comparisons test (0.01<*P<0.1, 0.001<**P<0.01, 0.0001<***P < 0.001, ****P<0.0001 vs control, and ns is not significant). n.d, not detected; n.t., non‐treat; WT, hIFN‐λ3‐WT; DE1, hIFN‐λ3‐DE1; G‐DE1, G‐hIFN‐λ3‐DE1.

Based on structural alignment within the IFN‐λ family (Figure S2, Supporting Information), we identified Asp35 in hIFN‐λ3 as a position analogous to Asn46 in hIFN‐λ1, a naturally glycosylated site (Figures S1a; S7a, Supporting Information).^62^ As this site lies distal from receptor‐binding interfaces, we reasoned that introducing N‐glycans here would minimize structural or functional disruption. To this end, we engineered D35N and K37S mutations to create an N‐X‐S glycosylation motif, generating a glycosylated variant termed G‐hIFN‐λ3‐DE1 (**Figure** **4**a). Expression in mammalian cells and subsequent purification confirmed glycan attachment to G‐hIFN‐λ3‐DE1, as evidenced by increased molecular weight and PNGaseF‐dependent band shifts (Figure 4b; S7b, Supporting Information). Importantly, G‐hIFN‐λ3‐DE1 maintained high thermostability (Tm = 90.2 °C), comparable to the non‐glycosylated hIFN‐λ3‐DE1 (Figure S7c, Supporting Information), indicating that the introduced glycan did not compromise protein folding. Moreover, glyco‐engineering markedly improved protein production yield, achieving a 2.5‐fold increase relative to wild‐type hIFN‐λ3 (Figure 4c), likely due to improved protein solubility conferred by the attached glycans.

To evaluate the functional impact of glycosylation on mucosal transport, we assessed the diffusion of G‐hIFN‐λ3‐DE1 through an artificial 3D gel system mimicking the nasal mucus composed of 1.2% hydroxyethyl cellulose (HEC), representing the viscous and glycan‐rich environment of the native mucus (1.6 Pa s^−1^).^[^ ^63^, ^64^ ^]^ Fluorescently labeled hIFN‐λ3‐WT, hIFN‐λ3‐DE1, and G‐hIFN‐λ3‐DE1 were applied atop a 1 mm‐thick HEC gel in a microplate, and penetration was examined by confocal microscopy after 10 min. G‐hIFN‐λ3‐DE1 exhibited markedly greater diffusion depth compared to both wild‐type and non‐glycosylated variants (Figure 4d), suggesting that the glycol‐engineering promoted more efficient diffusion of G‐hIFN‐λ3‐DE1 through the mucus‐like barrier.

We further assessed the biological relevance of mucosal diffusion by comparing *Mx1* mRNA induction in HNEpCs treated with hIFN‐λ3 variants (hIFN‐λ3‐WT, hIFN‐λ3‐DE1, G‐hIFN‐λ3‐DE1) directly or via the HEC gel placed in trans‐well inserts. Direct treatment of HNEpCs with each variant resulted in comparable levels of *Mx1* transcription, confirming that glycosylation did not affect intrinsic bioactivity (Figure 4e, left). In contrast, in the trans‐well system with 500 µm‐thickness of HEC gels, G‐hIFN‐λ3‐DE1 induced significantly higher *Mx1* expression in HNEpCs relative to hIFN‐λ3‐WT and hIFN‐λ3‐DE1 (Figure 4e, right). These results demonstrate that site‐specific glyco‐engineering improves mucosal penetration and functional delivery of IFN‐λ3 across mucus‐like barriers, offering a promising strategy for nasal protein drug development.

### 2.6 Prophylactic Antiviral Effects of Designed hIFN‐λ3 Against Respiratory Viruses In Vitro

**Figure 5:**
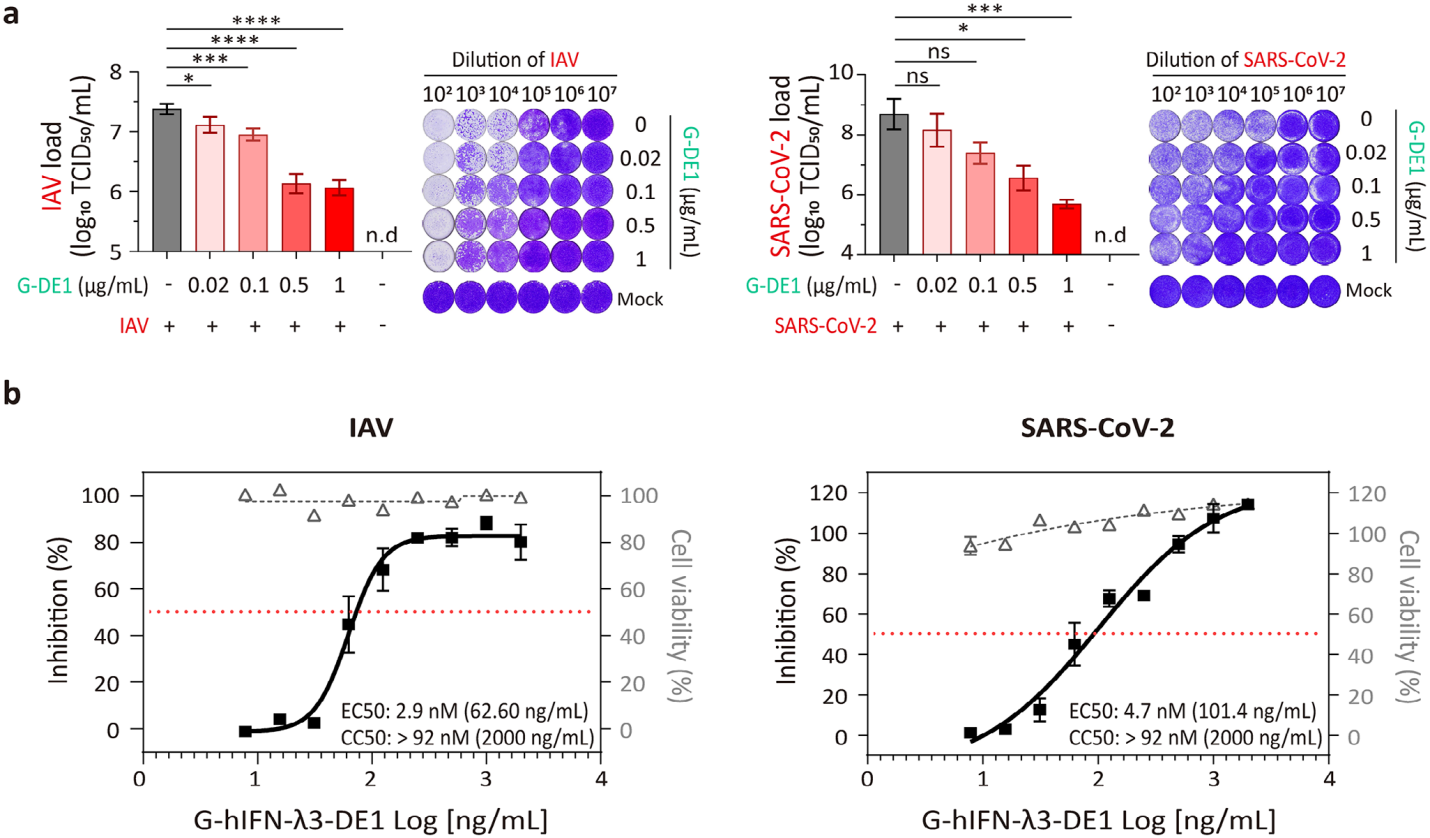
Prophylactic antiviral activity of G‐hIFN‐λ3‐DE1 against respiratory viruses in vitro. a) 50% tissue culture infectious dose (TCID_50_) assay against influenza A virus (IAV, H1N1 A/Puerto Rico/8/1934; PR8) and SARS‐CoV‐2 (Wuhan strain) with or without G‐hIFN‐λ3‐DE1 (G‐DE1) pretreatment (0.02–1 µg mL^−1^). Cells were infected with six serial 10‐fold virus dilutions (10^2^ to 10^7^). At 48 h post‐infection, cytopathic effects (CPE using crystal‐violet staining) were imaged, and TCID_50_ value were calculated by the Reed and Muench method^[^ ^66^ ^]^ (mean ± SD; *n* = 4 for IAV, *n* = 5 for SARS‐CoV‐2; left). Representative CPE images at each dilution are shown (right). b) Dose‐response of G‐DE1 against IAV and SARS‐CoV‐2 under a fixed inoculum. MDCK and Vero E6 cells were pretreated for 24 h with two‐fold serial dilutions of G‐DE1 (7.8–2000 ng mL^−1^), then infected with IAV (10^3.4^ TCID_50_/mL; 10^4^‐fold virus dilution) and SARS‐CoV‐2 (10^3.7^ TCID_50_/mL; 10^5^‐fold virus dilution), respectively (mean ±SEM; *n* = 3). Antiviral activity is plotted as inhibition of virus‐induced cell death (squares, left y‐axis), while viability of uninfected cells is shown to assess G‐DE1 cytotoxicity (open triangles, right y‐axis). The red dotted line denotes 50% inhibition of virus‐induced cell death. Statistical signifiance was determined by one‐way ANOVA followed by Tukey's multiple comparisons test (0.01<*P<0.1, 0.0001<***P<0.001, ****P<0.0001 versus control, n.d is not detected and ns is not significant). Curves are four‐parameter logistic fits (GraphPad Prism 8.0) with R^2^ = 0.939 (IAV) and 0.952 (SARS‐CoV‐2). EC_50_ and CC_50_ values are indicated in each panel.

To evaluate the broad‐spectrum prophylactic potential of engineered hIFN‐λ3, we assessed the antiviral activity of G‐hIFN‐λ3‐DE1 against respiratory mucosal viruses, including influenza A virus (IAV) and SARS‐CoV‐2. For in vitro evaluation, MDCK and Vero E6 cells were pretreated with G‐hIFN‐λ3‐DE1 (0.02–1 µg mL^−1^) for 24 h prior to viral infection. Antiviral effects were assessed by evaluating cytopathic effects (CPEs) at 48 h post‐infection, and 50% tissue culture infectious dose (TCID_50_) values were calculated by the Reed‐Muench method.^[^ ^65^, ^66^ ^]^ The untreated controls showed robust viral replication with TCID_50_ values exceeding 10^7^ for IAV and 10^8^ for SARS‐CoV‐2, whereas G‐hIFN‐λ3‐DE1 pretreatment inhibited replication of both viruses (**Figure** **5**a). Notably, pretreatment with 0.5 and 1 µg/mL G‐hIFN‐λ3‐DE1 significantly reduced virus‐induced cell death compared with untreated controls, and even the lowest dose of G‐hIFN‐λ3‐DE1 (0.02 µg/mL) conferred measurable antiviral activity against IAV.

We next explored the dose‐responsiveness of G‐hIFN‐λ3‐DE1 under fixed viral inoculum (IAV, 10^3.4^ TCID_50_/mL, 10^4^‐fold virus dilution; SARS‐CoV‐2, 10^3.7^ TCID_50_/mL, 10^5^‐fold virus dilution). G‐hIFN‐λ3‐DE1 was titrated as two‐fold serial dilutions (7.8–2000 ng mL^−1^), and antiviral activity was quantified by MTS measurement of virus‐induced cell death. The resulting curves were canonical sigmoid across the tested range (Figure 5b), yielding EC_50_ = 2.9 nM (Hill slope = 2.941) for IAV and EC_50_ = 4.7 nM (Hill slope = 0.8404) for SARS‐CoV‐2. Although the antiviral dose‐response for SARS‐CoV‐2 was gradual, this did not reflect poor pathway engagement, as G‐hIFN‐λ3‐DE1 induced robust, dose‐dependent ISG expression (*Isg15, Mx1, and Oas1*) with typical saturation kinetics (Figure S8, Supporting Information). Instead, the attenuated antiviral response against SARS‐CoV‐2 may be related to the interferon evasion mechanisms of SARS‐CoV‐2.^[^ ^67^ ^]^ Parallel viability assays in uninfected cells showed no cytotoxicity of G‐hIFN‐λ3‐DE1 up to 2000 ng/mL (CC_50_ > 92 nM). These findings demonstrate that G‐hIFN‐λ3‐DE1 effectively inhibits the replication of respiratory viruses in vitro, thereby supporting its potential as a prophylactic agent against a broad spectrum of respiratory viruses.

### 2.7 Intranasal Delivery of Designed hIFN‐λ3 Confers Effective Prophylactic Protection Against IAV Infection in Mice

**Figure 6:**
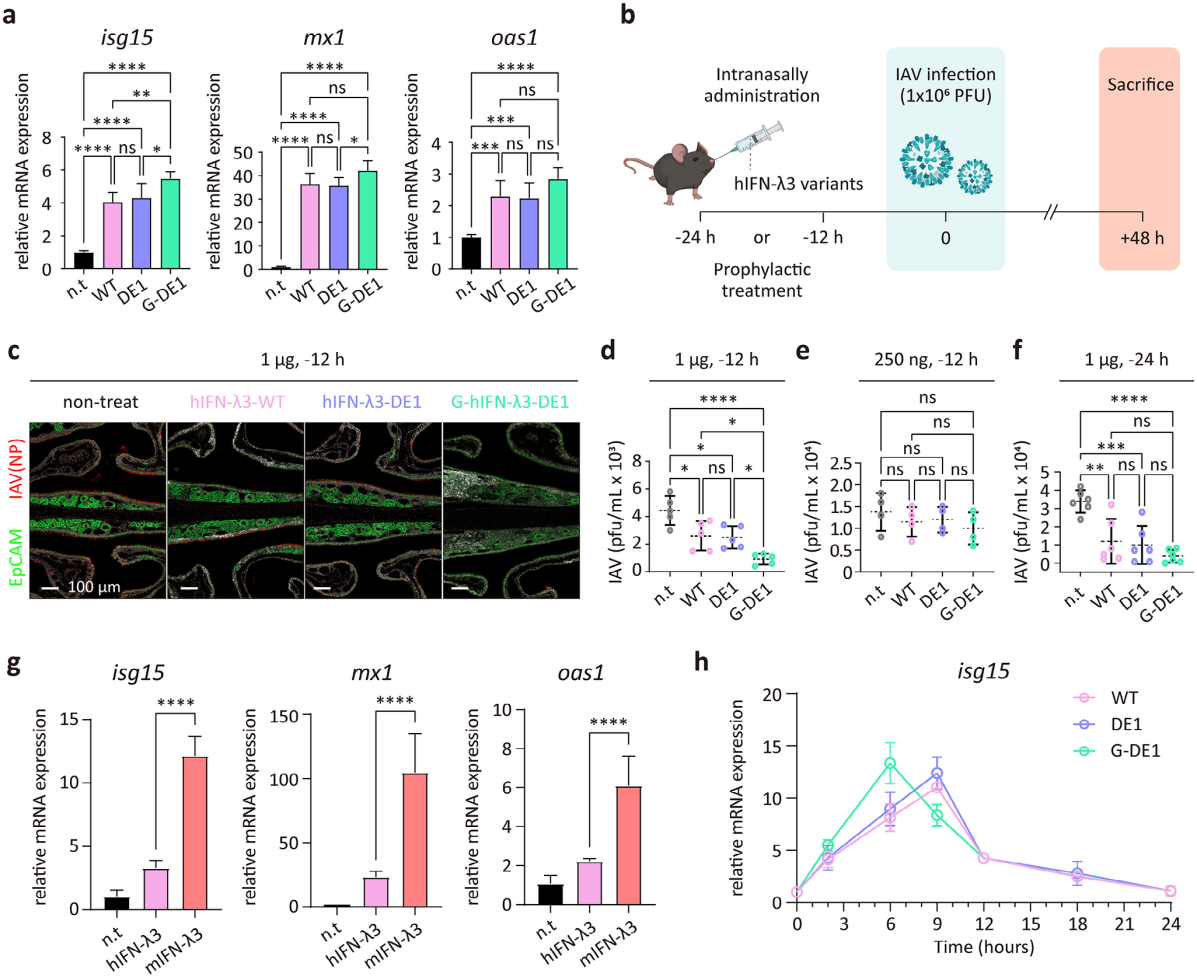
Intranasal delivery of G‐hIFN‐λ3‐DE1 provides rapid and enhanced prophylactic effects in an IAV‐infected mouse model. a) Relative expression levels of representative ISGs (*isg15, mx1, and oas1*) in mouse nasal turbinates 2 h after intranasal administration of recombinant hIFN‐λ3‐WT, hIFN‐λ3‐DE1, or G‐hIFN‐λ3‐DE1 (5 µg in 10 µL PBS; 0.5 mg mL^−1^). mRNA levels were analyzed by RT‐qPCR (*n* = 3), normalized to mouse *gapdh*, and expressed relative to non‐treated controls. b) Schematic experimental design for IAV infection and intranasal administration of hIFN‐λ3 variants (WT, DE1, and G‐DE1). Mice were intranasally pre‐treated with hIFN‐λ3 variants (250 ng or 1 µg; 10 µL) 12 h or 24 h prior to IAV infection (1 × 10⁶ PFU), followed by analysis 48 h post‐infection. c) Immunofluorescence images of nasal tissue sections of IAV‐infected mice (dpi=2) intranasally treated with hIFN‐λ3 variants (1ug of WT, DE1, and G‐DE1; 12 h pre‐dose → virus infection → 48 h readout). Epithelial cells were labeled with anti‐EpCAM antibody (green) and influenza A nucleoprotein was detected with anti‐IAV antibody (red). Representative images are shown for each treatment group. d–f) Quantification of viral titers in nasal tissues at 2 dpi by standard plaque assay under the indicated dose and treatment regimens (*n* = 5 mice per group for d, *n* = 4 for e, and *n* = 6 for f). Each dot represents an individual mouse; horizontal lines indicate mean ± SD. g) Relative expression levels of representative ISGs (*isg15*, *mx1*, and *oas1*) in mouse nasal turbinate 2 h after intranasal administration of recombinant human or mouse IFN‐λ3 WT (5 µg in 10 µL PBS; 0.5 mg mL^−1^). mRNA levels were analyzed by RT‐qPCR (n = 5), normalized to mouse gapdh, and expressed relative to non‐treated controls. h) Time‐course analysis of *isg15* expression in mouse nasal turbinate following intranasal administration of recombinant hIFN‐λ3‐WT, hIFN‐λ3‐DE1, or G‐hIFN‐λ3‐DE1 (5 µg in 10 µL PBS; 0.5 mg mL^−1^). Nasal tissues were collected at the indicated time points (0–24 h), and mRNA levels were quantified by RT‐qPCR (*n* = 3), normalized to mouse gapdh, and expressed relative to non‐treated controls. All data represent mean ± SD from independent experiments. Statistical significance was determined by one‐way ANOVA test followed by Tukey's multiple comparisons test (0.01<*P<0.1, 0.001<**P<0.01, 0.0001<***P < 0.001, ****P<0.0001 vs control and ns is not significant). n.t., non‐treat; WT, hIFN‐λ3‐WT; DE1, hIFN‐λ3‐DE1; G‐DE1, G‐hIFN‐λ3‐DE1.

We first examined the ability of the hIFN‐λ3 variants to induce interferon‐stimulated genes (ISGs) in vivo following intranasal administration. Mice treated with purified hIFN‐λ3‐WT, hIFN‐λ3‐DE1, or G‐hIFN‐λ3‐DE1 exhibited significant upregulation of *isg15*, *mx1*, and *oas1* transcripts in the nasal epithelium compared to untreated controls (**Figure** **6**a). Consistent with in vitro assay with HNEpCs, hIFN‐λ3‐WT and hIFN‐λ3‐DE1 induced comparable levels of ISG expression, suggesting that protein stabilization did not alter basal activity. In contrast, G‐hIFN‐λ3‐DE1 elicited slightly higher *isg15* expression than both non‐glycosylated variants, along with a modest increase in *mx1* and *oas1* expression. These results support the notion that glyco‐engineering enhances access to epithelial receptors beneath the mucus layer, potentially improving mucosal signal transduction and antiviral efficacy.

Next, we evaluated the prophylactic effect of three variants in a mouse model of influenza A virus (IAV) infection by assessing viral replication in the nasal epithelium. Mice received 1 µg of hIFN‐λ3‐WT, hIFN‐λ3‐DE1, or G‐hIFN‐λ3‐DE1 intranasally 12 h prior to viral challenge (Figure 6b). At 48 h post‐infection, IAV replication in the nasal cavity was visualized by confocal microscopy using IAV nucleoprotein (NP) staining (Figure 6c). The untreated group showed widespread NP signal (red) throughout the nasal epithelium (green), indicating extensive viral proliferation. However, mice treated with hIFN‐λ3‐WT or hIFN‐λ3‐DE1 exhibited moderate reductions in NP signal, while G‐hIFN‐λ3‐DE1 treatment led to a pronounced suppression of viral replication (Figure 6c). To quantitatively confirm these observations, plaque assays were performed using nasal tissue homogenates. Viral titers were significantly reduced in the G‐hIFN‐λ3‐DE1‐treated group, whereas moderate reductions were observed in mice treated with hIFN‐λ3‐WT or hIFN‐λ3‐DE1 (Figure 6d).

Interestingly, reduction of the intranasal dose from 1 µg to 250 ng abolished detectable prophylaxis (Figure 6e). We hypothesized that this reflected limited cross‐species potency of human IFN‐λ3 variants in mice rather than a lack of activity per se. Indeed, intranasal dosing elicited substantially weaker ISG induction in nasal tissue with human IFN‐λ3 than with mouse IFN‐λ3 (Figure 6g). Therefore, 1 µg of human IFN‐λ3 appears to be the minimal dose needed to produce measurable antiviral effects in this model, whereas the effective dose for mouse IFN‐λ3 is likely lower.

Additionally, administering 1 µg intranasal dose 24 h before viral challenge (24 h pre‐dose → infection → 48 h readout) minimized the apparent prophylactic differences among variants (Figure 6f), while G‐hIFN‐λ3‐DE1 showed a trend toward greater reduction, likely due to the saturation of interferon‐stimulated pathways. Nevertheless, the time‐course analysis of nasal ISG induction after treatment with hIFN‐λ3 variants (hIFN‐λ3‐WT, hIFN‐λ3‐DE1, or G‐hIFN‐λ3‐DE1) showed that G‐hIFN‐λ3‐DE1 elicited a more rapid and potent ISG response than hIFN‐λ3‐WT and hIFN‐λ3‐DE1. For instance, *isg15* expression peaked at 6 h with G‐hIFN‐λ3‐DE1, whereas peaks for hIFN‐λ3‐WT and hIFN‐λ3‐DE1 occurred later at 9 h (Figure 6h). By 12–24 h, ISG levels converged across groups (Figure 6h; Figure S9, Supporting Information). This kinetic profile explains why the enhanced mucosal penetration and local exposure of G‐hIFN‐λ3‐DE1 confers superior protection at the 12‐h window, whereas the subsequent pathway saturation by 24 h leads to the comparable outcomes observed at that later time point. Accordingly, a 12 h pre‐treatment with 1 µg of G‐hIFN‐λ3‐DE1 appears optimal for demonstrating the benefit of enhanced mucosal penetration and rapid onset of protection in this mouse model, underscoring the importance of optimizing dose and regimen for potential clinical translation.

Taken together, these findings demonstrate that glyco‐engineered G‐hIFN‐λ3‐DE1 exhibits a rapid onset of in vivo prophylactic protection against IAV infection when administered intranasally. This enhanced efficacy is likely due to improved diffusion through the viscous, glycan‐rich nasal mucus layer, enabling more efficient access to epithelial receptors and thereby enhancing antiviral signaling. These properties highlight the therapeutic potential of G‐hIFN‐λ3‐DE1 as a stable and effective prophylactic biologic for respiratory virus infections.

### 2.8 In Silico Prediction of Immunogenicity for G‐hIFN‐λ3‐DE1

To evaluate the immunogenic potential of the engineered G‐hIFN‐λ3‐DE1 variant, we performed an in silico analysis using DeepNeo‐v2,^[^ ^68^ ^]^ a deep learning–based tool that integrates MHC binding affinity and T cell reactivity prediction. Peptides spanning the engineered regions were assessed against a panel of representative human MHC class I and II alleles with broad global population coverage, including HLA‐A*02:01, A*24:02, DQB1*03:01, DQB1*03:02, and DQB1*05:01^[^ ^69^ ^]^ (**Table** **2**).

**Table 2:** In silico prediction of MHC‐binding and T cell‐reactive peptides in hIFN‐λ3‐WT and G‐hIFN‐λ3‐DE1.

hIFN‐λ3‐WT				G‐hIFN‐λ3‐DE1			
MHC class I (Allele)	Peptide [9‐mer]	MHC binding	TCR reactivity	MHC class I (Allele)	Peptide [9‐mer]	MHC binding	TCR reactivity
HLA‐A*02:01	LEESLLLK[D]	0.8629	0.6873	HLA‐A*02:01	EESLLLK[NC]	0.6302	0.5767
	LLK[DCK]CRS	0.6584	0.6546				
	RACI(QPQPT)	0.5915	0.8287		(TPEQREEAQ)	0.7547	0.5878
	(QPQPTAGPR)	0.5119	0.7563				
HLA‐A*24:01	–	–	–	HLA‐A*24:01	–	–	–
MHC class II (Allele)	Peptide (15‐mer)	MHC binding	TCR reactivity	MHC class II (Allele)	Peptide (15‐mer)	MHC binding	TCR reactivity
HLA‐DQB1*03:01	–	–	–	HLA‐DQB1*03:01	–	–	–
HLA‐DQB1*03:02	–	–	–	HLA‐DQB1*03:02	–	–	–
HLA‐DQB1*05:01	–	–	–	HLA‐DQB1*05:01	–	–	–

( ) Designed part [ ] Glyco‐engineering part.

For MHC class I, four peptides derived from the wild‐type and two from G‐hIFN‐λ3‐DE1 were predicted as potentially immunogenic only in the context of HLA‐A*02:01. Notably, the number of positive hits was low in both cases, and the associated scores for G‐hIFN‐λ3‐DE1 peptides were not significantly higher than those of the wild‐type. Furthermore, for MHC class II alleles, no peptides from either the wild‐type or DE1 variant exceeded the immunogenicity threshold (Table 2). These results suggest that the engineered modifications in G‐hIFN‐λ3‐DE1 do not significantly increase predicted immunogenicity. Nonetheless, a comprehensive immunogenicity assessment including *ex vivo* PBMC or T cell activation assays, repeat‐dose studies in animal preclinical models, and anti‐drug antibody (ADA) detection in clinical trials,^[^ ^70^ ^]^ will be essential to fully ensure long‐term safety. If immunogenic hotspots are identified through these studies, targeted sequence modifications (“de‐immunization”) can be implemented to further reduce T cell reactivity and enhance the translational potential of the candidate.

## 3 Discussion

In this study, we applied AI‐based protein design and glyco‐engineering to enhance the fitness of human interferon‐λ3 (hIFN‐λ3). The engineered hIFN‐λ3 variant combines enhanced biophysical properties, efficient mucosal diffusion, and more rapid onset of in vivo antiviral efficacy, supporting its potential as an intranasal prophylactic against respiratory viruses.

Recent advances in AI, such as AlphaFold2/3 and RFdiffusion, have enabled near‐experimental accuracy in structure prediction and have made *de novo* protein with functionally tailored folds, marking a paradigm shift that enables the active design of synthetic proteins including enzymes, binders, and therapeutics—previously inaccessible to classical bioengineering approaches. The current AI toolkit for protein research is rapidly expanding beyond structure prediction and design. Deep learning models for protein–ligand binding affinity prediction accelerate virtual small‐molecule drug screening and lead prioritization.^[^ ^71^ ^]^ Other AI‐based tools can now predict functional sites, such as post‐translational modification or protein–protein interaction interfaces, directly from sequence or structure, aiding in the rational engineering of protein function.^[^ ^72^, ^73^ ^]^ Moreover, sequence generation networks increasingly incorporate thermodynamic stability and solubility predictions, further improving both the fitness and developability of *de novo* binders and drug candidates.^[^ ^41^, ^74^ ^]^

A major advance of this work is the integration of AI‐based backbone reconfiguration with targeted surface patch engineering to address long‐standing challenges in protein stability. Flexible loops susceptible to proteolysis and solvent‐exposed hydrophobic patches are well‐known features that compromise protein stability and integrity. However, conventional engineering approaches have been limited in addressing these elements, largely due to the inherent difficulty of precisely modifying backbone conformations in a desired manner. Strategic sequence redesign and backbone remodeling—altering the structural framework of a protein—has been shown to significantly enhance thermostability and protease resistance.^[^ ^75^ ^]^ In this study, we also employed state‐of‐the‐art AI‐based protein design tools, including RFdiffusion,^[^ ^38^ ^]^ ProteinMPNN^[^ ^41^ ^]^ and AlphaFold2,^[^ ^32^ ^]^ to replace the α3‐4 loop in hIFN‐λ3 with a *de novo* α‐helix. This loop‐to‐helix transformation not only eliminated a thrombin cleavage motif, but also created a new, well‐packed hydrophobic core by shielding a surface‐exposed hydrophobic patch that had rendered the wild‐type protein unstable. Additional proline substitutions at formerly flexible sites further rigidified the local structure and reduced conformational entropy. As a result, the engineered hIFN‐λ3‐DE1 exhibits exceptional thermostability (melting temperature >90 °C) and robust resistance to proteolytic degradation, surpassing the stability achievable by conventional point mutations. These enhancements enabled the hIFN‐λ3‐DE1 to retain structural integrity and full antiviral potency even after prolonged thermal stress (eg. 2 weeks at 50 °C), highlighting its suitability for long‐term storage at ambient temperature without refrigeration. Our findings demonstrate how AI‐based backbone reconfiguration and hydrophobic patch shielding can be synergistically applied to dramatically enhance protein fitness, establishing a broadly applicable framework for designing stable and effective protein therapeutics for harsh biological environments. Since AI‐based prediction tools such as AlphaFold2/3 may have limitations in accurately predicting local structural changes caused by insertions, deletions, or point mutations, and also face challenges in capturing conformational dynamics and the effects of environmental factors,^[^ ^76^, ^77^, ^78^ ^]^ high‐resolution structural studies by X‐ray crystallography or cryo‐EM will be required to provide more definitive molecular details of our engineered hIFN‐λ3 in complex with its receptors.

In general, glyco‐engineering is a well‐established strategy that can simultaneously enhance a protein's stability, pharmacokinetics, and in vivo efficacy.^[^ ^79^ ^]^ A notable success of therapeutic biologic is darbepoetin‐α (Aranesp), a hyper‐glycosylated erythropoietin analog engineered with two extra N‐glycans, which demonstrated a ≈3‐fold increase in serum half‐life over regular EPO, enabling less frequent dosing.^[^ ^80^ ^]^ Similarly, glyco‐engineered interferon‐β variants—with additional N‐linked glycosylation—retained full receptor activity while exhibiting enhanced structural stability and systemic pharmacokinetic profiles.^[^ ^54^ ^]^ To our knowledge, our work provides the first evidence that glycan modification can accelerate mucus penetration following intranasal delivery. In particular, G‐hIFN‐λ3‐DE1 diffused more efficiently through a nasal mucus‐mimicking artificial gel system than its non‐glycosylated counterpart—likely due to the favorable physicochemical properties—demonstrating improved mucosal penetration, a key requirement for intranasal prophylaxis against respiratory viral infections. These enhancements translated into a more rapid onset of in vivo prophylaxis against influenza A virus (IAV) following intranasal administration, particularly within 12 h pre‐treatment window in mouse model. This strategy aligns with previous findings that hydrophilic moieties such as PEG or glycans can increase solubility and bioavailability. However, compared to post‐expression modifications like PEGylation, the genetically encoded glycan offers a more streamlined approach to tuning pharmacokinetics and mucosal interactions without additional processing steps. Moreover, it is well known that glycan heterogeneity is a key determinant of therapeutic protein quality, as it can affect efficacy, pharmacokinetics, and safety.^[^ ^81^, ^82^ ^]^ Future studies will be needed to analyze and optimize glycan composition of G‐hIFN‐λ3‐DE1 for consistent and maximal benefit, especially in the context of intranasal delivery.

Of note, introduction of *de novo* N‐linked glycan at a site distant from receptor‐binding interfaces improved protein production and solubility, making the G‐hIFN‐λ3‐DE1 variant well‐suited for scalable manufacturing. In this study, we selected the D35N/K37S site for *de novo* N‐glycan introduction based on detailed structural alignment within the IFN‐λ family. Specifically, Asp35 in hIFN‐λ3 corresponds to Asn46 in hIFN‐λ1—a naturally glycosylated site positioned distal from receptor‐binding interfaces—making it a rational and low‐risk engineering target. While this strategy proved effective on the first attempt, we recognize that additional sites may be considered for future optimization. For example, Cys38 in hIFN‐λ3 aligns with Asn61 in hIFN‐λ4, another naturally glycosylated position (Figure S3, Supporting Information), and may offer alternative opportunities for glycoengineering. Our previous work also showed that the P73N mutation in IFN‐λ4 resulted in efficient N‐glycosylation and significantly increased protein expression without compromising biological function.^[^ ^29^ ^]^ Notably, this site aligns with R50 in hIFN‐λ3, suggesting that engineering an N‐glycosylation motif at R50 may be another viable strategy. However, due to the small size of hIFN‐λ3 (≈22 kDa) and the replacement of the α3‐4 loop with the DE‐α4 helix, the number of accessible loop regions of hIFN‐λ3‐DE1 suitable for glycosylation is inherently limited. Thus, while further optimization is possible, the range of promising glycoengineering sites in hIFN‐λ3‐DE1 is likely to be narrower than in larger or less structurally constrained proteins. Collectively, these results position glyco‐engineering as a robust and versatile strategy for developing scalable, bioavailable, and functionally optimized nasal prophylactic biologics, complementing the stability gains achieved through AI‐based backbone reconfiguration.

An additional strength of our approach lies in the immunological properties of IFN‐λ3, which have been increasingly recognized as highly favorable for mucosal antiviral defense. Unlike type I interferons, which can induce broad systemic immune activation, type III interferons act primarily on epithelial cells due to the restricted expression of the IFN‐λ receptor complex (IFNLR1/IL‐10Rβ), thereby confining their activity to barrier tissues such as the respiratory tract.^[^ ^83^, ^84^ ^]^ This selective action enables potent antiviral signaling with minimal off‐target inflammation. In support of this, intranasally administered IFN‐λ2 has been shown to confer robust protection against multiple SARS‐CoV‐2 variants, including Omicron, in murine models.^[^ ^25^ ^]^ Notably, such protection was achieved without inducing pro‐inflammatory cytokines or damaging lung tissue, underscoring the tissue‐protective and inflammation‐sparing nature of IFN‐λ responses. In these models, IFN‐λ acted predominantly on radio‐resistant, non‐hematopoietic epithelial cells and accelerated viral clearance while preserving epithelial integrity.^[^ ^6^, ^85^ ^]^ Moreover, systemic administration of pegylated IFN‐λ1 in clinical trials has demonstrated antiviral efficacy comparable to type I IFNs, but with significantly fewer adverse effects,^[^ ^17^ ^]^ further supporting the safety profile of this cytokine class. Beyond its direct antiviral effects, IFN‐λ plays a crucial role in enhancing adaptive immunity. Mice lacking IFN‐λ signaling showed impaired CD8^+^ T cell and antibody responses following infection with a live‐attenuated influenza virus.^[^ ^86^ ^]^ Moreover, intranasal administration of IFN‐λ alongside subunit vaccines resulted in a 15‐fold increase in serum IgG level and mucosal IgA production, markedly improving survival after challenge with virulent influenza strains. Notably, this IgG‐boosting effect of IFN‐λ was not observed when the vaccine was delivered via intraperitoneal or subcutaneous routes, highlighting the critical role of IFN‐λ in potentiating adaptive immunity at mucosal surfaces. Building on these insights, our glyco‐engineered hIFN‐λ3 variant is designed for intranasal delivery to maximize localized action while minimizing systemic exposure. To reduce the risk of immunogenicity, we limited sequence alterations to a small region—specifically, 12 amino acids for structural stabilization and 2 for glycan addition—and designed the protein to be effective at low doses. While anti‐drug antibody responses cannot be entirely ruled out, the minimal modifications and tissue‐localized mechanism of action are expected to reduce such risks. Taken together, our findings reinforce the growing consensus that IFN‐λ is an ideal candidate for mucosal immunoprophylaxis, offering targeted and durable protection against diverse respiratory virus with minimal inflammatory consequences.

Our engineered hIFN‐λ3, developed through a dual design strategy of backbone stabilization and glycan modification, shows strong potential as an intranasal prophylactic agent against diverse respiratory viruses. As our in vivo evaluation showed certain limitations inherent to the current mouse model such as cross‐species potency constraints, the next steps toward clinical translation will include rigorous preclinical safety assessments and proof‐of‐concept efficacy studies in humans with particular attention to immunogenicity, dosing, pharmacokinetics, and treatment regimen to confirm its protective effects and tolerability. Successfully developing this biologic could introduce a new approach to respiratory virus prophylaxis by providing a fast‐acting, broadly protective shield at the site of infection, thereby strengthening our defenses against future respiratory pandemics.

## 4 Experimental Section/Methods

### Computational Design Process

RFdiffusion^[^ ^38^ ^]^ was employed to design new scaffolds to replace the flexible loop in hIFN‐λ3, which contains the thrombin cleavage consensus sequence, with a stable α‐helix for concealing the nearby exposed hydrophobic patch. In brief, the B chain of the crystal structure of hIFN‐λ3 (PDB code: 3HHC)^[^ ^49^ ^]^ was used as the input structure. RFdiffusion with was tasked building 10–14 residues to replace the loop connecting α3 and α4 (the α3‐4 loop, residues 105–116, L = 12) (Figure S3, Supporting Information). Three hydrophobic residues (Val59, Leu118, and Leu122), located at the center of the solvent‐exposed hydrophobic patch, were designated as spatial hotspots to guide backbone generation by RFdiffusion. Twenty‐five backbones replacing the α3‐4 loop of hIFN‐λ3 were generated at each diffusion step (T = 50, 100, 150, and 200) with the final contig being ‘B4‐104/10‐14/B117‐163′, resulting in a total of 100 designed hIFN‐λ3s. Next, hIFN‐λ3 candidates was selected designed containing a rigid α‐helix with more than two turns (> 8 amino acids) at the initial α3‐4 loop position. To do so, this was analyzed their secondary structures with the DSSP algorithm,^[^ ^39^, ^40^ ^]^ which identifies repetitive patterns of intra‐backbone hydrogen bonds by calculating the electrostatic energy based on the distance between the carbonyl group atoms (C, O) and the amide group atoms (N, H) of the protein backbone. Following the selection of 45 designed hIFN‐λ3 candidates, 10 sequences per designed backbone were generated with ProteinMPNN,^[^ ^41^ ^]^ keeping the original sequence of other hIFN‐λ3 parts fixed (sampling temperature of 0.1 and backbone noise of 0). Subsequently, their structures were predicted using AlphaFold2^[^ ^32^ ^]^ with the single sequence option. Structural templating with multiple sequence alignments (MSAs) was not used for structure prediction. A total of 450 designs with backbone and sequence redesign at the α3‐4 loop of hIFN‐λ3 were filtered to Cα RMSD ≤ 0.8 and pLDDT ≥ 93.0, resulting in 221 designed hIFN‐λ3 candidates.

To further select the final candidates with lower energy by minimizing the exposure of hydrophobic amino acids, the relative solvent accessibility (RSA) value was used,^[^ ^42^, ^43^ ^]^ which represents the exposure of amino acids by comparing the solvent‐accessible surface area (SASA) at the designated area to their maximum exposure (Max SASA^[^ ^43^ ^]^), and the total energy score calculated by energy functions^[^ ^44^, ^45^ ^]^ in Rosetta.^[^ ^87^ ^]^ First, the RSA was calculated value for the hotspot residues (Val59, Leu118, and Leu122), the hydrophobic amino acids at the center of the exposed hydrophobic patch. The 221 designs was filtered to those with an RSA of hotspots ≤ 0.075 (average value among the 221 outputs), resulting in 90 designs. Next, the solvent exposure of the hydrophobic amino acids (Ala, Gly, Val, Ile, Leu, Phe, and Met) was calculated on the newly designed α‐helix (RSA of hydrophobic residues in DE‐α4) among the resulting 90 designs. Lastly, the structures of 90 designs was refined using the relax option^[^ ^46^, ^47^ ^]^ in Rosetta^[^ ^87^ ^]^ and calculated the total energy score using the “ref2015” (Rosetta energy function).^[^ ^44^, ^45^ ^]^ Consequently, 90 designs were filtered with RSA of hydrophobic residues in DE‐α4’ ≤ 0.1 and total energy ≤ −477 kcal mol^−1^, resulting in five final designed hIFN‐λ3 candidates (hIFN‐λ3‐DE1, 2, 3, 4, and 5).

The multi‐metric filtering strategy used here is widely employed in state‐of‐the‐art AI‐based *de novo* protein design workflows to effectively identify structurally reliable, conformationally accurate, and biophysically stable design candidates prior to experimental validation.^[^ ^88^, ^89^, ^90^ ^]^ Each metric employed captures a distinct biophysical property critical for successful protein design. Specifically, the pLDDT score from AlphaFold2 assesses local structural reliability at the residue level, with high values (≥90) indicating accurate backbone and side‐chain modeling, thus effectively eliminating low‐confidence or poorly folded designs.^[^ ^76^, ^90^, ^91^ ^]^ Cα RMSD quantifies structural fidelity by comparing backbone conformations of RF diffusion‐designed scaffolds and their AlphaFold2‐predicted models after sequence optimization, ensuring the preservation of intended conformational topology.^[^ ^89^, ^90^ ^]^ Relative solvent accessibility (RSA) measures residue solvent exposure and identifies destabilizing exposed hydrophobic regions. Since the key objective of this study was to shield an exposed hydrophobic patch with the *de novo*‐designed DE‐α4 helix, RSA was the most crucial metric for selecting variants with minimized surface hydrophobicity, thereby promoting protein solubility and stability. Finally, the Rosetta total energy score provides a thermodynamic stability estimation based on an energy function that incorporates *van der Waals* interactions, electrostatics, hydrogen bonding, and solvation effects. Lower Rosetta total energy scores guided this selection toward energetically favorable and structurally robust designs.^[^ ^92^ ^]^ As these metrics reflect inherently different biophysical properties, integrating them into a single weighted scoring scheme would be challenging and potentially misleading. Therefore, each metric independently and sequentially was applied, initially filtering by pLDDT and RMSD to ensure structural accuracy and conformational reliability, followed by RSA and Rosetta total energy to refine stability and solubility.

### Cell Lines and Cell Culture

HNEpC cells (C‐12620, PromoCell) were cultured in Airway Epithelial Cell Growth Medium (C‐21060, PromoCell) at 37 °C in a humidified 5% CO2 incubator. Expi 293‐F cells (A14527, Thermo Fisher Scientific) were maintained in Expi293 Expression Medium (A1435102, Thermo Fisher Scientific) under shaking conditions at 37 °C in a humidified 8% CO_2_ incubator.

### Constructs for Recombinant hIFN‐λs and Designed hIFN‐λ3 Protein Expression

Full‐length human IFN‐λ1, ‐λ2, and ‐λ3, along with designed hIFN‐λ3 variants, were cloned into the HindIII and NotI sites of a modified pcDNA3.1 vector (#V79020, Invitrogen) incorporating a TEV protease cleavage site and C‐terminal 6×His tag. Glycoengineered human IFN‐λ4 (ehIFN‐λ4: L28N, P73N),^[^ ^29^ ^]^ ectodomain of hIFN‐λR1 and hIL‐10Rβ was cloned into the HindIII and XbaI sites of the modified pcDNA3.1 vector (#V79020, Invitrogen) containing the thrombin cleavage sequence and Protein A tag.

### Expression and Purification of Recombinant hIFN‐λs and Designed hIFN‐λ3 Candidates

Recombinant plasmids (1 µg) were transfected into 2.5 × 106 Expi293‐F cells (#A14527, Thermo Fisher Scientific) using the ExpiFectamine 293 Transfection kit (#A14524, Thermo Fisher Scientific). Cells were cultured at 37 °C and 8% CO_2_ with shaking (orbital shaker, 120 rpm) for 4 days. After centrifugation to remove the cells, the supernatants (hIFN‐λ1, 2, and 3 and designed hIFN‐λ3 candidates) were loaded onto the Ni‐NTA agarose affinity column (#30210, QIAGEN). After washes with 10 column volumes of wash buffer (PBS, 25 mm imidazole), proteins were eluted with elution buffer (PBS, 250 mm imidazole). Eluted proteins were treated with TEV protease (1% [v/v]) at room temperature for 2 h to remove the C‐terminal His tag. For purifying the ehIFN‐λ4, the supernatants were loaded onto IgG Sepharose 6 Fast Flow (#17‐0969‐01, Cytiva). After washes with 10 column volumes of wash buffer (PBS), the Protein A─fused eIFN‐λ4─bound resin was incubated with thrombin (1% [v/v] in PBS) at 4 °C for 16 h to remove the C‐terminal Protein A tag. After incubation, proteins were eluted with an elution buffer (PBS). All of eluted recombinant proteins were further purified by size‐exclusion chromatography using a Superose 6 Increase 10/300 GL column (#GE29‐0915‐96, Cytiva) or Superdex 200 Increase 10/300 GL column (#GE28‐9909‐44, Cytiva) equilibrated with PBS. The peak fractions were pooled and concentrated to ≈1 mg mL^−1^ using an Amicon Ultra centrifugal filter (#UFC8010, Millipore). For deglycosylation analysis, hIFN‐λs were incubated with GST‐PNGaseF (10 µg mL^−1^) at 37 °C for 3 h.

### Immunoblotting of Intracellular Signal Activation in HNEpCs

HNEpC cells (#C‐12620, PromoCell) were cultured in basal Airway Epithelial cell Growth Medium (#C‐21060, PromoCell) without supplement mix for starvation at 37 °C in a humidified 5% CO_2_ incubator for 12 h. After starvation, the cells were incubated with 100 ng mL^−1^ of recombinant hIFN‐λs for 1 h. Cells were then washed with cold PBS and lysed with lysis buffer (10 mm Tris‐Cl pH 7.4, 150 mm NaCl, 5 mm EDTA, 10% glycerol, 1% Triton X‐100, protease inhibitor, phosphatase inhibitor). Lysates were mixed with a 5× SDS sample buffer containing 2‐mercaptoethanol and heated at 90 °C for 5 min. Samples were electrophoresed on a 10% SDS protein gel, and transferred to a 0.45‐µm nitrocellulose membrane. The membrane was blocked by incubation with 5% (w/v) skim milk in Tris‐buffered saline containing 0.1% Tween‐20 at room temperature for 1 h and then incubated with Stat1 (D1K9Y) rabbit monoclonal antibody (mAb; 1:1000 dilution, #14994, Cell Signaling), or phospho‐Stat1 (Y701) rabbit mAb (1:1000 dilution, #9167, Cell Signaling), or beta actin (C4) mouse mAb (1:1000 dilution, #sc‐47778, Santa Cruz) at 4 °C for 8 h. Blots were then incubated with horseradish peroxidase (HRP)‐conjugated goat anti‐rabbit IgG antibody (1:5000 dilution, #12‐348, Sigma‐Aldrich) or HRP‐conjugated goat anti‐mouse IgG antibody (1:5000 dilution, #HAF007, R&D Systems). Immunoreactive Stat1, phosphor‐Stat1 and beta actin were visualized using enhanced chemiluminescence. Signal intensities were quantified using ImageJ (v1.54) and GraphPad Prism (v8.0.2) software.

### RT‐qPCR of Representative ISGs in HNEpCs and Vero E6 Cells

For ISG induction in HNEpCs (#C‐12620, PromoCell), starved cells were incubated with 100 ng mL^−1^ of recombinant hIFN‐λs (wild type, designed and heat‐incubated proteins) for 12 h. For dose‐dependent ISG induction in Vero E6 cells, cells were starved with serum‐free MEM (#LM007‐08, Welgene) for 12 h and then incubated with G‐hIFN‐λ3‐DE1 (7.8–2000 ng mL^−1^; 4‐fold serial dilution) for 24 h. Following incubation for both protocols, total RNA was extracted using Trizol reagent (#15596026, Invitrogen) following the manufacturer's protocol. Extracted RNA was reverse‐transcribed using an oligo‐dT primer and SuperScript III First‐strand Synthesis System (#18080‐051, Invitrogen) according to the kit instructions. Next, qPCR was performed using PowerUP SYBR Green Master Mix (#A25741, Applied Biosystems) on a QuantStudio 5 Real‐Time PCR System (Applied Biosystems). The RT‐qPCR primers are listed in Table S1 (Supporting Information). Ct values were normalized against *18S rRNA* (for HNEpCs) or *Gapdh* (for Vero E6 cells), and the relative mRNA expression levels compared to untreated samples (PBS) were calculated using the ΔΔCt method (fold expression = 2^–ΔΔCt^). Statistical significance for time‐course qPCR was determined using one‐way ANOVA.

### Thermal Shift Assay for Measuring Tm

Thermal shift assays were conducted using the QuantStudio 5 Real‐Time PCR System (Applied Biosystems) and the Protein Thermal Shift Dye Kit (#4461146, Applied Biosystems). Each 20 µL reaction contained 12.5 µL of protein (1 mg/mL), 2.5 µL of diluted Protein Thermal Shift Dye (final concentration: 8×), and 5 µL of Protein Thermal Shift Buffer. Samples were loaded in a 96‐well PCR plate and subjected to a two‐step melt curve: step 1, 25 °C for 2 min; step 2, temperature increase at 0.05 °C s^−1^ to 99 °C, then held at 99 °C for 2 min. The filter was set at ROX with no quencher and no passive filter. Representative fluorescence spectra from ≥3 independent experiments were plotted. Tm values were determined by differentiating melt curves in GraphPad Prism 8.0.

### Thrombin Susceptibility Test

Purified hIFN‐λ3‐WT and hIFN‐λ3‐DE1 were incubated with thrombin (1% [v/v] in PBS) at 37 °C for 1 h. Samples then were incubated at 99 °C for 5 min with 5× SDS sample buffer containing a 2‐mercaptoethanol and electrophoresed on a 4%–20% Mini‐PROTEAN TGX Precast Protein Gel (4561094, Bio‐Rad).

### CD Spectra for Analyzing Secondary Structure

The circular dichroism (CD) spectra of hIFN‐λ3‐WT and hIFN‐λ3‐DE1 (1 mg mL^−1^, 60 µL) were recorded at 25 °C using a Jasco CD J‐815 150‐L spectropolarimeter (JASCO) in a demountable U‐shaped cuvette with a 0.2‐mm path length. Measurements were taken from 195 to 260 nm with 1‐nm step intervals, and each spectrum was averaged from three scans. Secondary structure contents were analyzed using BeStSel^[^ ^93^ ^]^ based on the spectra between 195 and 260 nm.

### Binding Kinetics Analysis using SPR

The binding kinetics of hIFN‐λ3‐WT and hIFN‐λ3‐DE1 to the receptor protein hIFN‐λR1 were measured using surface plasmon resonance (SPR) on a Biacore T200 system (GE healthcare). The Series S sensor chip NTA (#BR100532, Cytiva) was used to immobilized the His‐tagged hIFN‐λ‐3‐WT or hIFN‐λ3‐DE1. The running buffer was 1X PBS‐P+ buffer containing 50 nM EDTA (#28995084, Cytiva). His‐tagged hIFN‐λ‐3‐WT or hIFN‐λ3‐DE1 was immobilized up to ≈200 RU. Next, analytes (recombinant ectodomain of hIFN‐λR1) diluted in running buffer, were applied over the immobilized sensor chips at 3 different concentrations (6.25, 12.5, and 25 nm) for 60 s at a flow rate of 10 µL min^−1^. The bound analytes were dissociated by washing with running buffer for 120 s. The equilibrium dissociation constant (K_D_) was calculated as the ratio of the dissociation rate constant (k_off_) to the association rate constant (k_on_). Kinetic parameters were obtained by global fitting to a 1:1 binding model using the Biacore Insight Evaluation Software.

### Protein Aggregation Test

For short‐term incubation, purified hIFN‐λ3‐WT and hIFN‐λ3‐DE1 (1 mg mL^−1^, 20 µL) were incubated at 50, 60, 70, 80, or 90 °C for 5 min. For long‐term incubation, the purified hIFN‐λ3‐WT and hIFN‐λ3‐DE1 (1 mg mL^−1^, 100 µL) were incubated at 45 or 50 °C for 2 weeks. The temperature was increased at 0.1 °C s^−1^ from room temperature (25 °C) to the target temperature. After incubation, samples were centrifuged at low speed (3,000 g, 10 min), and the protein concentration in the supernatant was quantified using a DS‐11+ Spectrophotometer (DeNovix).

### HEC Gel Diffusion Assay; 3D Imaging

For 3D imaging of diffusion in the HEC gel systems, a 1.2% hydroxyethyl cellulose (HEC) solution was prepared by dissolving 0.6 g of HEC powder (#09368, Sigma‐Aldrich) in 50 mL of distilled water while heating and stirring at 70 °C. For 3D imaging, 150 µL of a 1 mm alkyne‐BDP‐FL solution (#CLK‐045, Jena Bioscience) was added into the 1.2% HEC solution, followed by coating the 24‐well culture plate (#30024, SPL Life Sciences). For synthesis hIFN‐λ3 variants‐AF647, hIFN‐λ3 variants were reacted with AF647‐NHS ester (#A37566, Thermo Fisher) at a molar ratio of 1:4 at room temperature for 2 h. Unreacted dye was removed using an Amicon Ultra‐0.5 centrifugal filter with a 3 kDa molecular weight cutoff (#UFC5003, Millipore). Then, 1 µg of AF647‐labeled hIFN‐λ3 variants was applied to the HEC gels and analyzed by confocal microscopy (LSM 880, Carl Zeiss). Z‐stack images were captured, and the fluorescence intensity across various focal planes was quantified using Zen Blue software (Carl Zeiss).

### HEC Gel Diffusion Assay; Trans‐Well Model

For the HEC gel diffusion assay in a trans‐well system, HNEpC cells (#C‐12620, PromoCell) in a 24‐well microplate were starved in basal medium for 12 h prior to stimulation. A 500 µm‐thick layer of 1.2% HEC solution was added to the trans‐well inserts (#36624, SPL Life Sciences) and placed in 24‐well microplates. hIFN‐λ3 variants (50 ng/5 µL) were loaded to the top of the gel in the insert and removed after 15 min incubation. After an additional 12 h incubation at 37 °C, total RNA from cells in the lower chamber was extracted using the TaKaRa MiniBEST Universal RNA Extraction Kit (#9767A, TaKaRa). qPCR was performed with PowerUP SYBR Green Master Mix (#A25741, Applied Biosystems) on a QuantStudio 5 Real‐Time PCR System (Applied Biosystems). The RT‐qPCR primers are listed in Table S1 (Supporting Information). Ct values were normalized to *18S rRNA*, and gene expression was calculated using the ΔΔCt method (fold expression = 2^–ΔΔCt^). One‐way ANOVA was used to determine statistical significance.

### In Vitro Antiviral Assay, TCID50

Madin‐Darby canine kidney cells (MDCK, #CCL‐34, ATCC) or African green monkey kidney cells (Vero E6, #CRL‐1586, ATCC) were seeded at a density of 2 × 10⁴ cells per well in 96‐well plates using complete Minimum Essential Medium (MEM, #LM007‐08, Welgene) for infection with influenza virus (H1N1 A/Puerto Rico/8/1934 or PR8) and the SARS‐CoV‐2 Wuhan (hCoV/Korea/KCDC03/2020), respectively. The cells were incubated overnight at 37 °C in a 5% CO_2_ atmosphere. The following day, the medium was replaced with serum‐free MEM containing G‐hIFN‐λ3‐DE1 at 1, 500, 100, or 20 ng mL^−1^, and the cells were incubated for an additional 24 h. After pre‐treatment, the cells were infected with serial 10‐fold dilutions (10^2^–10⁷) of each viruses in plain MEM. After 1 h of viral adsorption at 37 °C for SARS‐CoV‐2 and 35 °C for influenza virus, the inoculum was removed and replaced with MEM supplemented with 2% fetal bovine serum (FBS, # 16000044, Gibco); for influenza virus infection, 1 µg mL^−1^ TPCK‐trypsin was also added. At 48 h post‐infection, the cells were washed with phosphate‐buffered saline (PBS) and stained with 0.1% crystal violet. Cytopathic effects were imaged, and TCID_50_ values were calculated using the Reed‐Muench method.^[^ ^66^ ^]^

### In Vitro Antiviral Assay, MTS Assay

Cells were prepared as described for the TCID_50_ assay using MDCK (for influenza A virus, H1N1 A/Puerto Rico/8/1934; PR8) and Vero E6 (for SARS‐CoV‐2 Wuhan strain, hCoV/Korea/KCDC03/2020) cells. Following overnight incubation, the medium was replaced with serum‐free MEM containing G‐hIFN‐λ3‐DE1, prepared in a two‐fold serial dilution series (9 concentrations, starting at 2000 ng mL^−1^), and the cells were incubated for an additional 24 h. After pre‐treatment, the cells were infected with IAV at 10^3.4^ TCID_50_/mL (10^4^‐fold dilution) or SARS‐CoV‐2 at 10^3.7^ TCID_50_/mL (10^5^‐fold dilution) in plain MEM (100 µL/well). After 1 h of viral adsorption at 37 °C for SARS‐CoV‐2 and 35 °C for influenza virus, the inoculum was removed and replaced with MEM supplemented with 2% fetal bovine serum (FBS, #16000044, Gibco); for influenza virus infection, 1 µg mL^−1^ TPCK‐trypsin was also added. At 48 h post‐infection, the cells were washed twice with phosphate‐buffered saline (PBS), followed by addition of 20 µL of MTS reagent (3‐(4,5‐dimethylthiazol‐2‐yl)‐5‐(3‐carboxymethoxyphenyl)‐ 2‐(4‐sulfophenyl)‐2H‐tetrazolium) (#G3580, Promega) and 30 µL MEM per well. After incubation for 1 h at 37 °C in a 5% CO_2_ atmosphere, absorbance was measured at 490 nm using a microplate reader (SpectraMax microplate reader, Molecular Devices). Normalized viability values were used to generate dose–response curves, and EC_50_ and Hill slope values were calculated by nonlinear regression with a four‐parameter logistic model using GraphPad Prism 8.0. In parallel, cytotoxicity of G‐hIFN‐λ3‐DE1 was assessed under identical conditions without viral infection.

### Animals

Six‐to‐eight‐week‐old male C57BL/6J mice were used and housed in a specific‐pathogen‐free (SPF) facility at the Korea Advanced Institute of Science and Technology (KAIST). All procedures used in this study complied with guidelines and protocol (KA2021‐004) of the Institutional Animal Care and Use Committee of KAIST.

### Virus Infection and hIFN‐λ3 Variants Treatment

For upper respiratory tract infection, mice were intranasally inoculated with 1 × 10^6^ plaque‐forming units (PFU) of influenza A/PR/8/34 virus (H1N1, provided by HK Lee, KAIST, South Korea) in a 16 µL volume under light anesthesia (4% isoflurane in oxygen). For intranasal hIFN‐λ3s treatment, mice were intranasally administered 250 ng or 1 µg of hIFN‐λ3‐WT, hIFN‐λ3‐DE1 or G‐hIFN‐λ3‐DE1 in 10 µL of PBS.

### Measurement of Mouse ISG Gene Expression at Nasal Tissue by RT‐qPCR

At the indicated time points post hIFN‐λ3 variant administration (2, 6, 9, 12,18, 21, or 24 h with 5ug dose), nasal turbinates were isolated and homogenized in Trizol (#15596026, Invitrogen). Total RNA was extracted using the RNeasy Mini Kit (#74106, QIAGEN) according to the manufacturer's protocol. cDNA was synthesized from 1 µg of RNA using an oligo‐dT primer and the SuperScript III First‐Strand Synthesis System for RT‐PCR (#18080‐051, Invitrogen). qPCR was performed using PowerUP SYBR Green Master Mix (#A25741, Applied Biosystems) on a QuantStudio 5 Real‐Time PCR System (Applied Biosystems). The RT‐qPCR primers are listed in Table S1 (Supporting Information). Ct values were normalized to Gapdh expression and relative gene expression was calculated using the ΔΔCt method (fold expression = 2^–ΔΔCt^).

### Immunofluorescence Imaging

For immunofluorescence staining, mice were perfused with ice‐cold PBS via the left ventricle to flush out blood, followed by fixation with 2% paraformaldehyde (PFA, #15710, EMS). For cryo‐sectioning of the mouse nasal cavity, heads were post‐fixed in 4% PFA overnight at 4 °C, decalcified in 0.5 M EDTA (#ML005‐01, Welgene) for 96 h at 4 °C, and dehydrated by submersion in 30% sucrose (#SUC01, LPS) for 48 h at 4 °C. Samples were embedded in Tissue‐Tek OCT compound (#4583, Sakura), frozen, and sectioned at 15 µm thickness. Frozen sections of nasal tissues were stained with 4′,6‐diamidino‐2‐phenylindole (DAPI, #D‐9542, Sigma‐Aldrich) and mounted in Fluoromount‐G (#0100‐01, SouthernBiotech). Primary antibodies (1:300) included anti‐influenza A (goat polyclonal, #ab20841, Abcam) and AF488‐conjugated anti‐mouse CD326 (Ep‐CAM) (rat monoclonal, #118210, BioLegend). Secondary antibody (1:1000) was Alexa Fluor 594 AffiniPure rabbit anti‐goat IgG (H+L) (#305‐585‐045, Jackson ImmunoResearch). Images were acquired using a confocal microscope with 10× or 20× objectives (LSM980, Zeiss).

### Influenza Virus Titration

At 2 days post infection, nasal turbinate tissue was homogenized in minimum essential medium containing 0.3% BSA using a Precellys 24 bead‐beating homogenizer (#P000669‐PR240‐A, Bertin Technologies) and lysing kit tubes (#P000911‐LYSK0‐A.O, Bertin Technologies). Homogenates were centrifuged (15 min, 2300 g) to clear debris, and viral titers in the supernatants were determined by standard plaque assay on Madin‐Darby canine kidney cells (MDCK, provided by HK Lee, KAIST), as previously described.^[^ ^94^ ^]^

### In silico Prediction of Immunogenicity

To assess the immunogenic potential of the engineered G‐hIFN‐λ3‐DE1 protein, an in silico immunogenicity analysis was performed using DeepNeo‐v2,^[^ ^68^ ^]^ web‐based deep learning platform (http://deepneo.net) that integrates MHC binding affinity prediction (DeepNeo‐mhc) and T cell reactivity evaluation (DeepNeo‐tcr) for both MHC class I and II alleles. DeepNeo accepts up to 100 amino acids per submission in Protein Mode. To ensure full coverage, both the wild‐type and G‐hIFN‐λ3‐DE1 sequences was divided into overlapping 100 amino acid segments using a sliding window with a step size of 50 amino acids (e.g., residues 1–100, 51–150, 101–196). This approach enabled thorough analysis of all possible 9‐mer (MHC I) and 15‐mer (MHC II) peptides, including those spanning segment boundaries. Each segment was analyzed against a representative panel of human MHC alleles selected for broad global population coverage,^[^ ^69^ ^]^ including HLA‐A*02:01 and HLA‐A*24:02 (MHC class I), and HLA‐DQB1*03:01, HLA‐DQB1*03:02, and HLA‐DQB1*05:01 (MHC class II). DeepNeo‐v2 also provides separate prediction scores (ranging from 0 to 1) for MHC binding and T cell reactivity. A peptide was considered potentially immunogenic only if both scores exceeded the default threshold of 0.5. Immunogenicity profiles were then compared between the wild‐type and engineered variant, with particular focus on peptides overlapping the modified regions in G‐hIFN‐λ3‐DE1.

### Statistical Analysis

Data were presented as mean ±standard deviation (SD) or ±standard error of the mean (SEM) for all experiments. Statistical analysis was performed to assess significant differences between experimental groups. The pre‐processing of data involved the normalization of mRNA expression values against housekeeping genes (e.g., human *18s rRNA*, mouse *gapdh* or green monkey *Gapdh*) for time‐course qPCR experiments and the normalization of protein band intensities against β‐actin as an internal control for immunoblotting experiments. For all experimental replicates, a sample size of *n* = 3 to 6 was used, as specified in the figure legends. Statistical analysis was performed using one‐way ANOVA followed by Tukey's or Sidak's multiple comparisons test and a P value of less than 0.1 was considered statistically significant (0.01<*P<0.1, 0.001<**P<0.01, 0.0001<***P< 0.001, ****P<0.0001 vs control). All statistical analyses were conducted using GraphPad Prism (v8.0.2) software.

## Conflict of Interest

The authors declare no conflict of interest.

## Author Contributions

J.Y., S.Y., and J.H.K. contributed equally to this work. J.Y. and H.M.K. designed the experiments and analyzed the data; J.Y. designed and purified the proteins, conducted protein stability tests, and performed biological activity experiments at the cellular level; S.Y. set up the nasal mucus mimic systems and conducted the protein diffusion assays; J.H.K. performed cross‐reactivity and antiviral activity experiments in IAV‐infected mouse models; L.F.V. wrote scripts for the computational protein design processes; J.H.C., M.R.C., K.B.K, K.D.K designed and conducted experiments to assess the antiviral activity of engineered proteins on SARS‐CoV‐2 and IAV infections at the cellular level; J.Y. and H.J.R. performed immunogenicity prediction of engineered proteins; J.Y., S.Y., J.H.K., L.F.V., J.H.C., M.R.C., K.B.K., K.D.K., M.C., H.J.C., J.E.O., and H.M.K. wrote the manuscript.

## Supporting information



Supporting Information

## Data Availability

The source data for all figures and supplementary figures are available as Source Data file. All the other data and materials used for the analysis are available from the corresponding author upon reasonable request. Source data are provided with this paper.

## References

[1] T. Kawai , S. Akira , Nat. Immunol. 2006, 7, 131. 16424890 10.1038/ni1303

[2] H. M. Lazear , T. J. Nice , M. S. Diamond , Immunity 2015, 43, 15. 26200010 10.1016/j.immuni.2015.07.001 PMC4527169

[3] D. Novick , B. Cohen , M. Rubinstein , Cell 1994, 77, 391. 8181059 10.1016/0092-8674(94)90154-6

[4] P. Sheppard , W. Kindsvogel , W. F. Xu , K. Henderson , S. Schlutsmeyer , T. E. Whitmore , R. Kuestner , U. Garrigues , C. Birks , J. Roraback , C. Ostrander , D. Dong , J. Shin , S. Presnell , B. Fox , B. Haldeman , E. Cooper , D. Taft , T. Gilbert , F. J. Grant , M. Tackett , W. Krivan , G. McKnight , C. Clegg , D. Foster , K. M. Klucher , Nat. Immunol. 2003, 4, 63. 12469119 10.1038/ni873

[5] S. V. Kotenko , G. Gallagher , V. V. Baurin , A. Lewis‐Antes , M. L. Shen , N. K. Shah , J. A. Langer , F. Sheikh , H. Dickensheets , R. P. Donnelly , Nat. Immunol. 2003, 4, 69. 12483210 10.1038/ni875

[6] J. Klinkhammer , D. Schnepf , L. Ye , M. Schwaderlapp , H. H. Gad , R. Hartmann , D. Garcin , T. Mahlakoiv , P. Staeheli , Elife 2018, 7. 10.7554/eLife.33354 PMC5953542 29651984

[7] M. Mordstein , E. Neugebauer , V. Ditt , B. Jessen , T. Rieger , V. Falcone , F. Sorgeloos , S. Ehl , D. Mayer , G. Kochs , M. Schwemmle , S. Günther , C. Drosten , T. Michiels , P. Staeheli , J. Virol. 2010, 84, 5670. 20335250 10.1128/JVI.00272-10 PMC2876583

[8] L. Ye , D. Schnepf , P. Staeheli , Nat. Rev. Immunol. 2019, 19, 614. 31201377 10.1038/s41577-019-0182-z

[9] I. E. Galani , V. Triantafyllia , E. E. Eleminiadou , O. Koltsida , A. Stavropoulos , M. Manioudaki , D. Thanos , S. E. Doyle , S. V. Kotenko , K. Thanopoulou , E. Andreakos , Immunity 2017, 46, 875. 28514692 10.1016/j.immuni.2017.04.025

[10] M. L. Stanifer , C. Kee , M. Cortese , C. M. Zumaran , S. Triana , M. Mukenhirn , H. G. Kraeusslich , T. Alexandrov , R. Bartenschlager , S. Boulant , Cell Rep. 2020, 32, 107863. 32610043 10.1016/j.celrep.2020.107863 PMC7303637

[11] Y. J. Jeon , C. H. Gil , A. Jo , J. Won , S. Kim , H. J. Kim , Antivir Res 2020, 180, 104860. 32565134 10.1016/j.antiviral.2020.104860 PMC7303047

[12] I. E. Galani , N. Rovina , V. Lampropoulou , V. Triantafyllia , M. Manioudaki , E. Pavlos , E. Koukaki , P. C. Fragkou , V. Panou , V. Rapti , O. Koltsida , A. Mentis , N. Koulouris , S. Tsiodras , A. Koutsoukou , E. Andreakos , Nat. Immunol. 2021, 22, 32. 33277638 10.1038/s41590-020-00840-x

[13] Y. Fukuda , T. Homma , H. Inoue , C. Onitsuka , H. Ikeda , Y. Goto , Y. Sato , T. Kimura , K. Hirai , S. Ohta , M. Yamamoto , S. Kusumoto , S. Suzuki , A. Tanaka , H. Sagara , J. Med. Virol. 2021, 93, 4559. 33811680 10.1002/jmv.26993 PMC8250710

[14] Y. Fukuda , T. Homma , H. Inoue , Y. Goto , Y. Sato , H. Ikeda , C. Onitsuka , H. Sato , K. Akimoto , T. Ebato , H. Suganuma , T. Kawahara , H. Mikuni , Y. Uchida , S. Suzuki , A. Tanaka , H. Sagara , Sci Rep‐Uk 2022, 12, 5458. 10.1038/s41598-022-09544-8 PMC8969403 35361913

[15] A. Wack , E. Terczynska‐Dyla , R. Hartmann , Nat. Immunol. 2015, 16, 802. 26194286 10.1038/ni.3212 PMC7096991

[16] Y. G. Liu , S. W. Jin , S. S. Zhang , T. J. Xia , Y. H. Liao , R. L. Pan , M. Z. Yan , Q. Chang , Front Immunol 2024, 15, 1338096. 38495892 10.3389/fimmu.2024.1338096 PMC10940417

[17] J. J. Feld , C. Kandel , M. J. Biondi , R. A. Kozak , M. A. Zahoor , C. Lemieux , S. M. Borgia , A. K. Boggild , J. Powis , J. McCready , D. H. S. Tan , T. Y. Chan , B. Coburn , D. Kumar , A. Humar , A. Chan , B. O'Neil , S. Noureldin , J. Booth , R. Hong , D. Smookler , W. Aleyadeh , A. Patel , B. Barber , J. Casey , R. Hiebert , H. Mistry , I. Choong , C. Hislop , D. M. Santer , et al., Lancet Resp Med 2021, 9, 498. 10.1016/S2213-2600(20)30566-X PMC7906707 33556319

[18] G. Reis , E. A. M. Silva , D. C. M. Silva , L. Thabane , V. H. S. Campos , T. S. Ferreira , C. V. Q. Santos , A. M. R. Nogueira , A. P. F. G. Almeida , L. C. M. Savassi , A. D. Figueiredo‐Neto , A. C. F. Dias , A. J. Freire , C. Bitares , A. C. Milagres , E. D. Callegari , M. I. C. Simplicio , L. B. Ribeiro , R. Oliveira , O. Harari , L. A. Wilson , J. I. Forrest , H. Ruton , S. Sprague , P. McKay , C. M. Guo , E. H. Limbrick‐Oldfield , S. Kanters , G. H. Guyatt , C. R. Rayner , et al., New Engl J Med 2023, 388, 518.36780676

[19] P. Jagannathan , J. R. Andrews , H. Bonilla , H. Hedlin , K. B. Jacobson , V. Balasubramanian , N. Purington , S. Kamble , C. R. de Vries , O. Quintero , K. Feng , C. Ley , D. Winslow , J. Newberry , K. Edwards , C. Hislop , I. Choong , Y. Maldonado , J. Glenn , A. Bhatt , C. Blish , T. Wang , C. Khosla , B. A. Pinsky , M. Desai , J. Parsonnet , U. Singh , Nat. Commun. 2021, 12. 10.1038/s41467-021-22177-1 PMC8009873 33785743

[20] W. S. T. Consortium , H. Pan , R. Peto , A. M. Henao‐Restrepo , M. P. Preziosi , V. Sathiyamoorthy , Q. Abdool Karim , M. M. Alejandria , C. Hernandez Garcia , M. P. Kieny , R. Malekzadeh , S. Murthy , K. S. Reddy , M. Roses Periago , P. Abi Hanna , F. Ader , A. M. Al‐Bader , A. Alhasawi , E. Allum , A. Alotaibi , C. A. Alvarez‐Moreno , S. Appadoo , A. Asiri , P. Aukrust , A. Barratt‐Due , S. Bellani , M. Branca , H. B. C. Cappel‐Porter , N. Cerrato , T. S. Chow , et al., N. Engl. J. Med. 2021, 384, 497.33264556

[21] Z. Zhou , L. Ren , L. Zhang , J. Zhong , Y. Xiao , Z. Jia , L. Guo , J. Yang , C. Wang , S. Jiang , D. Yang , G. Zhang , H. Li , F. Chen , Y. Xu , M. Chen , Z. Gao , J. Yang , J. Dong , B. Liu , X. Zhang , W. Wang , K. He , Q. Jin , M. Li , J. Wang , Cell Host Microbe 2020, 27, 883. 32407669 10.1016/j.chom.2020.04.017 PMC7196896

[22] A. Park , Cell Host Microbe 2020, 27, 870. 32464097 10.1016/j.chom.2020.05.008 PMC7255347

[23] H. Shin , S. Kim , A. Jo , J. Won , C. H. Gil , S. Y. Yoon , H. Cha , H. J. Kim , Frontiers in Immunology 2022, 13. 10.3389/fimmu.2022.1009424 PMC9744928 36524125

[24] C. H. Gil , C. Oh , J. Lee , M. Jang , J. Han , S. D. Cho , S. H. Park , J. H. Park , H. J. Kim , Acs Appl Mater Inter 2024, 16, 11147. 10.1021/acsami.3c13677 38407048

[25] Z. L. Chong , C. E. Karl , P. J. Halfmann , Y. Kawaoka , E. S. Winkler , S. P. Keeler , M. J. Holtzman , J. S. Yu , M. S. Diamond , Cell Rep. 2022, 39. 10.1016/j.celrep.2022.110799 PMC9021357 35523172

[26] A. G. Beule , GMS Curr Top Otorhinolaryngol Head Neck Surg 2010, 9, Doc07. 22073111 10.3205/cto000071 PMC3199822

[27] S. Gizurarson , Biol. Pharm. Bull. 2015, 38, 497. 25739664 10.1248/bpb.b14-00398

[28] Y. Ozsoy , S. Gungor , E. Cevher , Molecules 2009, 14, 3754. 19783956 10.3390/molecules14093754 PMC6254717

[29] J. H. Chung , S. H. Hong , N. Seo , T. S. Kim , H. J. An , P. Lee , E. C. Shin , H. M. Kim , Cytokine 2020, 125, 154833. 31479875 10.1016/j.cyto.2019.154833 PMC7129780

[30] M. Syedbasha , J. Linnik , D. Santer , D. O'Shea , K. Barakat , M. Joyce , N. Khanna , D. L. Tyrrell , M. Houghton , A. Egli , J Vis Exp 2016. 10.3791/53575 PMC4828978 27023275

[31] C. Dellgren , H. H. Gad , O. Hamming , J. Melchjorsen , R. Hartmann , Genes & Immunity 2009, 10, 125. 18987645 10.1038/gene.2008.87

[32] J. Jumper , R. Evans , A. Pritzel , T. Green , M. Figurnov , O. Ronneberger , K. Tunyasuvunakool , R. Bates , A. Zidek , A. Potapenko , A. Bridgland , C. Meyer , S. A. A. Kohl , A. J. Ballard , A. Cowie , B. Romera‐Paredes , S. Nikolov , R. Jain , J. Adler , T. Back , S. Petersen , D. Reiman , E. Clancy , M. Zielinski , M. Steinegger , M. Pacholska , T. Berghammer , S. Bodenstein , D. Silver , O. Vinyals , et al., Nature 2021, 596, 583. 34265844 10.1038/s41586-021-03819-2 PMC8371605

[33] J. Y. Chang , Eur. J. Biochem. 1985, 151, 217. 2863141 10.1111/j.1432-1033.1985.tb09091.x

[34] D. B. Wigley , A. R. Clarke , C. R. Dunn , D. A. Barstow , T. Atkinson , W. N. Chia , H. Muirhead , J. J. Holbrook , Bioch. Biophys. Acta 1987, 916, 145. 10.1016/0167-4838(87)90221-4 3663683

[35] A. A. Pakula , R. T. Sauer , Nature 1990, 344, 363. 2314475 10.1038/344363a0

[36] C. Strub , C. Alies , A. Lougarre , C. Ladurantie , J. Czaplicki , D. Fournier , BMC Biochem. 2004, 5, 9. 15251041 10.1186/1471-2091-5-9 PMC479692

[37] M. M. Gromiha , Biopolymers 2009, 91, 591. 19283830 10.1002/bip.21187

[38] J. L. Watson , D. Juergens , N. R. Bennett , B. L. Trippe , J. Yim , H. E. Eisenach , W. Ahern , A. J. Borst , R. J. Ragotte , L. F. Milles , B. I. M. Wicky , N. Hanikel , S. J. Pellock , A. Courbet , W. Sheffler , J. Wang , P. Venkatesh , I. Sappington , S. V. Torres , A. Lauko , V. De Bortoli , E. Mathieu , S. Ovchinnikov , R. Barzilay , T. S. Jaakkola , F. Dimaio , M. Baek , D. Baker , Nature 2023, 620, 1089. 37433327 10.1038/s41586-023-06415-8 PMC10468394

[39] R. P. Joosten , T. A. te Beek , E. Krieger , M. L. Hekkelman , R. W. Hooft , R. Schneider , C. Sander , G. Vriend , Nucleic Acids Res. 2011, 39, D411. 21071423 10.1093/nar/gkq1105 PMC3013697

[40] W. Kabsch , C. Sander , Biopolymers 1983, 22, 2577. 6667333 10.1002/bip.360221211

[41] J. Dauparas , I. Anishchenko , N. Bennett , H. Bai , R. J. Ragotte , L. F. Milles , B. I. M. Wicky , A. Courbet , R. J. de Haas , N. Bethel , P. J. Y. Leung , T. F. Huddy , S. Pellock , D. Tischer , F. Chan , B. Koepnick , H. Nguyen , A. Kang , B. Sankaran , A. K. Bera , N. P. King , D. Baker , Science 2022, 378, 49. 36108050 10.1126/science.add2187 PMC9997061

[42] M. Z. Tien , A. G. Meyer , D. K. Sydykova , S. J. Spielman , C. O. Wilke , PLoS One 2013, 8, 80635. 10.1371/journal.pone.0080635 PMC3836772 24278298

[43] S. Miller , J. Janin , A. M. Lesk , C. Chothia , J. Mol. Biol. 1987, 196, 641. 3681970 10.1016/0022-2836(87)90038-6

[44] H. Park , P. Bradley , P. Greisen , Y. Liu , V. K. Mulligan , D. E. Kim , D. Baker , F. DiMaio , J. Chem. Theory Comput. 2016, 12, 6201. 27766851 10.1021/acs.jctc.6b00819 PMC5515585

[45] R. F. Alford , A. Leaver‐Fay , J. R. Jeliazkov , M. J. O'Meara , F. P. DiMaio , H. Park , M. V. Shapovalov , P. D. Renfrew , V. K. Mulligan , K. Kappel , J. W. Labonte , M. S. Pacella , R. Bonneau , P. Bradley , R. L. Dunbrack , R. Das , D. Baker , B. Kuhlman , T. Kortemme , J. J. Gray , J. Chem. Theory Comput. 2017, 13, 3031. 28430426 10.1021/acs.jctc.7b00125 PMC5717763

[46] L. G. Nivon , R. Moretti , D. Baker , PLoS One 2013, 8, 59004. 10.1371/journal.pone.0059004 PMC3614904 23565140

[47] P. Conway , M. D. Tyka , F. DiMaio , D. E. Konerding , D. Baker , Protein Sci. 2014, 23, 47. 24265211 10.1002/pro.2389 PMC3892298

[48] J. Abramson , J. Adler , J. Dunger , R. Evans , T. Green , A. Pritzel , O. Ronneberger , L. Willmore , A. J. Ballard , J. Bambrick , S. W. Bodenstein , D. A. Evans , C. C. Hung , M. O'Neill , D. Reiman , K. Tunyasuvunakool , Z. Wu , A. Zemgulyte , E. Arvaniti , C. Beattie , O. Bertolli , A. Bridgland , A. Cherepanov , M. Congreve , A. I. Cowen‐Rivers , A. Cowie , M. Figurnov , F. B. Fuchs , H. Gladman , R. Jain , et al., Nature 2024, 630, 493. 38718835 10.1038/s41586-024-07487-w PMC11168924

[49] H. H. Gad , C. Dellgren , O. J. Hamming , S. Vends , S. R. Paludan , R. Hartmann , J. Biol. Chem. 2009, 284, 20869. 19457860 10.1074/jbc.M109.002923 PMC2742852

[50] J. L. Mendoza , W. M. Schneider , H. H. Hoffmann , K. Vercauteren , K. M. Jude , A. Xiong , I. Moraga , T. M. Horton , J. S. Glenn , Y. P. de Jong , C. M. Rice , K. C. Garcia , Immunity 2017, 46, 379. 28329704 10.1016/j.immuni.2017.02.017 PMC5510750

[51] A. M. Sinclair , S. Elliott , J. Pharm. Sci. 2005, 94, 1626. 15959882 10.1002/jps.20319

[52] Y. Liu , A. Nguyen , R. L. Wolfert , S. Q. Zhuo , Biotechnol Progr 2009, 25, 1468. 10.1002/btpr.241 19637381

[53] N. Ceaglio , M. Etcheverrigaray , R. Kratje , A. Oggero , Biochimie 2008, 90, 437. 18039474 10.1016/j.biochi.2007.10.013

[54] K. Song , I. S. Yoon , N. A. Kim , D. H. Kim , J. Lee , H. J. Lee , S. Lee , S. Choi , M. K. Choi , H. H. Kim , S. H. Jeong , W. S. Son , D. D. Kim , Y. K. Shin , PLoS One 2014, 9, 96967. 10.1371/journal.pone.0096967 PMC4032242 24858932

[55] R. J. Goodson , N. V. Katre , Site‐Directed Pegylation of Recombinant Interleukin‐2 at Its Glycosylation Site, Bio‐Technol 1990, 8, 343. 10.1038/nbt0490-343 1366535

[56] C. Araman , R. E. Thompson , S. Wang , S. Hackl , R. J. Payne , C. F. Becker , Chem. Sci. 2017, 8, 6626. 28989689 10.1039/c7sc02719b PMC5625290

[57] K. R. Santhanakrishnan , J. Koilpillai , D. Narayanasamy , Cureus 2024, 16, 66669. 10.7759/cureus.66669 PMC11390148 39262507

[58] S. K. Lai , D. E. O'Hanlon , S. Harrold , S. T. Man , Y. Y. Wang , R. Cone , J. Hanes , Proc Natl Acad Sci USA 2007, 104, 1482. 17244708 10.1073/pnas.0608611104 PMC1785284

[59] J. T. Huckaby , S. K. Lai , Adv Drug Deliv Rev 2018, 124, 125. 28882703 10.1016/j.addr.2017.08.010

[60] C. S. Schneider , Q. Xu , N. J. Boylan , J. Chisholm , B. C. Tang , B. S. Schuster , A. Henning , L. M. Ensign , E. Lee , P. Adstamongkonkul , Sci. Adv. 2017, 3, 1601556. 10.1126/sciadv.1601556 PMC5381952 28435870

[61] C. Y. Bao , Q. Zhang , Eur. Polym. J. 2019, 112, 263.

[62] Z. J. Miknis , E. Magracheva , W. Li , A. Zdanov , S. V. Kotenko , A. Wlodawer , J. Mol. Biol. 2010, 404, 650. 20934432 10.1016/j.jmb.2010.09.068 PMC2991516

[63] Y. Majima , T. Harada , T. Shimizu , K. Takeuchi , Y. Sakakura , S. Yasuoka , S. Yoshinaga , Am J. Respir. Crit. Care Med. 1999, 160, 421. 10430708 10.1164/ajrccm.160.2.9805117

[64] H. Viswanathan , I. A. Brownlee , J. P. Pearson , S. Carrie , American journal of rhinology 2006, 20, 554. 17063754 10.2500/ajr.2006.20.2935

[65] P. G. Madonov , V. A. Svyatchenko , S. S. Legostaev , N. A. Kikhtenko , A. A. Kotlyarova , L. A. Oleinik , G. I. Baikalov , Bull. Exp. Biol. Med. 2021, 172, 53. 34791556 10.1007/s10517-021-05330-0 PMC8598276

[66] R. Lj , Am J Hyg 1938, 27, 493.

[67] L. Miorin , T. Kehrer , M. T. Sanchez‐Aparicio , K. Zhang , P. Cohen , R. S. Patel , A. Cupic , T. Makio , M. H. Mei , E. Moreno , K. M. White , R. Rathnasinghe , M. Uccellini , S. Y. Gao , T. Aydillo , I. Mena , X. Yin , L. Martin‐Sancho , N. J. Krogan , S. K. Chanda , M. Schotsaert , R. W. Wozniak , Y. Ren , B. R. Rosenberg , B. M. A. Fontoura , A. García‐Sastre , P Natl Acad Sci, USA 2020, 117, 28344. 10.1073/pnas.2016650117 PMC7668094 33097660

[68] J. Y. Kim , H. Bang , S. J. Noh , J. K. Choi , Nucleic Acids Res. 2023, 51, W134. 37070174 10.1093/nar/gkad275 PMC10320182

[69] A. Sanchez‐Mazas , J. M. Nunes , E. A. Dominguez , P. Gerbault , N. K. Faye , W. Almawi , M. Andreani , E. Arrieta‐Bolanos , D. G. Augusto , S. Buhler , Best Practice & Research Clinical Haematology 2024, 37, 101559. 39098805 10.1016/j.beha.2024.101559

[70] V. Jawa , F. Terry , J. Gokemeijer , S. Mitra‐Kaushik , B. J. Roberts , S. Tourdot , A. S. De Groot , Front Immunol 2020, 11, 1301. 32695107 10.3389/fimmu.2020.01301 PMC7338774

[71] H. Zhang , X. Liu , W. Cheng , T. Wang , Y. Chen , Comput. Biol. Med. 2024, 174, 108435. 38608327 10.1016/j.compbiomed.2024.108435

[72] P. Gainza , F. Sverrisson , F. Monti , E. Rodola , D. Boscaini , M. M. Bronstein , B. E. Correia , Nat. Methods 2020, 17, 184. 31819266 10.1038/s41592-019-0666-6

[73] Y. J. Jang , Q. Q. Qin , S. Y. Huang , A. T. J. Peter , X. M. Ding , B. Kornmann , Nat. Commun. 2024, 15, 6601. 39097570 10.1038/s41467-024-50955-0 PMC11297950

[74] H. Dieckhaus , M. Brocidiacono , N. Z. Randolph , B. Kuhlman , Proc Natl Acad Sci, USA 2024, 121, 2314853121. 10.1073/pnas.2314853121 PMC10861915 38285937

[75] K. H. Sumida , R. Nunez‐Franco , I. Kalvet , S. J. Pellock , B. I. M. Wicky , L. F. Milles , J. Dauparas , J. Wang , Y. Kipnis , N. Jameson , A. Kang , J. De La Cruz , B. Sankaran , A. K. Bera , G. Jimenez‐Oses , D. Baker , J. Am. Chem. Soc. 2024, 146, 2054. 38194293 10.1021/jacs.3c10941 PMC10811672

[76] M. Akdel , D. E. V. Pires , E. P. Pardo , J. Janes , A. O. Zalevsky , B. Meszaros , P. Bryant , L. L. Good , R. A. Laskowski , G. Pozzati , A. Shenoy , W. Zhu , P. Kundrotas , V. R. Serra , C. H. M. Rodrigues , A. S. Dunham , D. Burke , N. Borkakoti , S. Velankar , A. Frost , J. Basquin , K. Lindorff‐Larsen , A. Bateman , A. V. Kajava , A. Valencia , S. Ovchinnikov , J. Durairaj , D. B. Ascher , J. M. Thornton , N. E. Davey , et al., Nat. Struct. Mol. Biol. 2022, 29, 1056. 36344848 10.1038/s41594-022-00849-w PMC9663297

[77] G. R. Buel , K. J. Walters , Nat. Struct. Mol. Biol. 2022, 29, 1. 35046575 10.1038/s41594-021-00714-2 PMC11218004

[78] P. Y. Lin , S. C. Huang , K. L. Chen , Y. C. Huang , C. Y. Liao , G. J. Lin , H. Lee , P. Y. Chen , Bot. Stud. 2025, 66, 14. 40402396 10.1186/s40529-025-00462-2 PMC12098255

[79] R. J. Sola , K. Griebenow , BioDrugs 2010, 24, 9. 20055529 10.2165/11530550-000000000-00000 PMC2805475

[80] S. Elliott , T. Lorenzini , S. Asher , K. Aoki , D. Brankow , L. Buck , L. Busse , D. Chang , J. Fuller , J. Grant , N. Hernday , M. Hokum , S. Hu , A. Knudten , N. Levin , R. Komorowski , F. Martin , R. Navarro , T. Osslund , G. Rogers , N. Rogers , G. Trail , J. Egrie , Nat. Biotechnol. 2003, 21, 414. 12612588 10.1038/nbt799

[81] G. Walsh , R. Jefferis , Nat. Biotechnol. 2006, 24, 1241. 17033665 10.1038/nbt1252

[82] R. Jefferis , Nat. Rev. Drug Discovery 2009, 8, 226. 19247305 10.1038/nrd2804

[83] T. Mahlakoiv , P. Hernandez , K. Gronke , A. Diefenbach , P. Staeheli , PLoS Pathog. 2015, 11, 1004782. 10.1371/journal.ppat.1004782 PMC4388470 25849543

[84] C. Sommereyns , S. Paul , P. Staeheli , T. Michiels , PLoS Pathog. 2008, 4, 1000017. 10.1371/journal.ppat.1000017 PMC2265414 18369468

[85] A. Broggi , F. Granucci , I. Zanoni , J. Exp. Med. 2020, 217, 20190295. 10.1084/jem.20190295 PMC7037241 31821443

[86] L. Ye , D. Schnepf , J. Becker , K. Ebert , Y. Tanriver , V. Bernasconi , H. H. Gad , R. Hartmann , N. Lycke , P. Staeheli , Nat. Immunol. 2019, 20, 593. 30886417 10.1038/s41590-019-0345-x

[87] A. Leaver‐Fay , M. Tyka , S. M. Lewis , O. F. Lange , J. Thompson , R. Jacak , K. Kaufman , P. D. Renfrew , C. A. Smith , W. Sheffler , I. W. Davis , S. Cooper , A. Treuille , D. J. Mandell , F. Richter , Y. E. Ban , S. J. Fleishman , J. E. Corn , D. E. Kim , S. Lyskov , M. Berrondo , S. Mentzer , Z. Popovic , J. J. Havranek , J. Karanicolas , R. Das , J. Meiler , T. Kortemme , J. J. Gray , B. Kuhlman , et al., Methods Enzymol 2011, 487, 545. 21187238 10.1016/B978-0-12-381270-4.00019-6 PMC4083816

[88] N. R. Bennett , B. Coventry , I. Goreshnik , B. Huang , A. Allen , D. Vafeados , Y. P. Peng , J. Dauparas , M. Baek , L. Stewart , F. DiMaio , S. De Munck , S. N. Savvides , D. Baker , Nat. Commun. 2023, 14, 2625. 37149653 10.1038/s41467-023-38328-5 PMC10163288

[89] S. L. Lisanza , J. M. Gershon , S. W. K. Tipps , J. N. Sims , L. Arnoldt , S. J. Hendel , M. K. Simma , G. Liu , M. Yase , H. Wu , C. D. Tharp , X. Li , A. Kang , E. Brackenbrough , A. K. Bera , S. Gerben , B. J. Wittmann , A. C. McShan , D. Baker , Nat. Biotechnol. 2024. 10.1038/s41587-024-02395-w PMC12339374 39322764

[90] A. Lauko , S. J. Pellock , K. H. Sumida , I. Anishchenko , D. Juergens , W. Ahern , J. Jeung , A. F. Shida , A. Hunt , I. Kalvet , C. Norn , I. R. Humphreys , C. Jamieson , R. Krishna , Y. Kipnis , A. Kang , E. Brackenbrough , A. K. Bera , B. Sankaran , K. N. Houk , D. Baker , Science 2025, 388, adu2454. 10.1126/science.adu2454 PMC12288761 39946508

[91] K. Tunyasuvunakool , J. Adler , Z. Wu , T. Green , M. Zielinski , A. Zidek , A. Bridgland , A. Cowie , C. Meyer , A. Laydon , S. Velankar , G. J. Kleywegt , A. Bateman , R. Evans , A. Pritzel , M. Figurnov , O. Ronneberger , R. Bates , S. A. A. Kohl , A. Potapenko , A. J. Ballard , B. Romera‐Paredes , S. Nikolov , R. Jain , E. Clancy , D. Reiman , S. Petersen , A. W. Senior , K. Kavukcuoglu , E. Birney , et al., Nature 2021, 596, 590. 34293799 10.1038/s41586-021-03828-1 PMC8387240

[92] J. K. Leman , B. D. Weitzner , S. M. Lewis , J. Adolf‐Bryfogle , N. Alam , R. F. Alford , M. Aprahamian , D. Baker , K. A. Barlow , P. Barth , B. Basanta , B. J. Bender , K. Blacklock , J. Bonet , S. E. Boyken , P. Bradley , C. Bystroff , P. Conway , S. Cooper , B. E. Correia , B. Coventry , R. Das , R. M. De Jong , F. DiMaio , L. Dsilva , R. Dunbrack , A. S. Ford , B. Frenz , D. Y. Fu , C. Geniesse , et al., Nat. Methods 2020, 17, 665. 32483333 10.1038/s41592-020-0848-2 PMC7603796

[93] A. Micsonai , E. Moussong , F. Wien , E. Boros , H. Vadaszi , N. Murvai , Y. H. Lee , T. Molnar , M. Refregiers , Y. Goto , A. Tantos , J. Kardos , Nucleic Acids Res. 2022, 50, W90. 35544232 10.1093/nar/gkac345 PMC9252784

[94] E. Kudo , E. Song , L. J. Yockey , T. Rakib , P. W. Wong , R. J. Homer , A. Iwasaki , Proc Natl Acad Sci, USA 2019, 116, 10905. 31085641 10.1073/pnas.1902840116 PMC6561219

