# Supplementary material for: Computational Design and Glycoengineering of Interferon‐Lambda for Nasal Prophylaxis Against Respiratory Viruses
Source: Adv Sci (Weinh). 2025 Nov 20;13(6):e06764. doi: 10.1002/advs.202506764 (PMC12866752; doi:10.1002/advs.202506764)
Supplement: Supplementary file 1 — Supporting Information [file ADVS-13-e06764-s001.pdf]

Supporting Information

Computational Design and Glycoengineering of Interferon-Lambda for Nasal Prophylaxis against Respiratory Viruses

Jeongwon Yun, Seungju Yang, Jae Hyuk Kwon, Luiz Felipe Vecchietti, Meeyoung Cha, Ji Hyun Choi, Mi-ra Choi, Keun Bon Ku, Hyun-Joo Ro, Kyun-Do Kim, Hyun Jung Chung, Ji Eun Oh*, and Ho Min Kim**

This PDF file includes:

Figure S1 to S9

Table S1 to S3

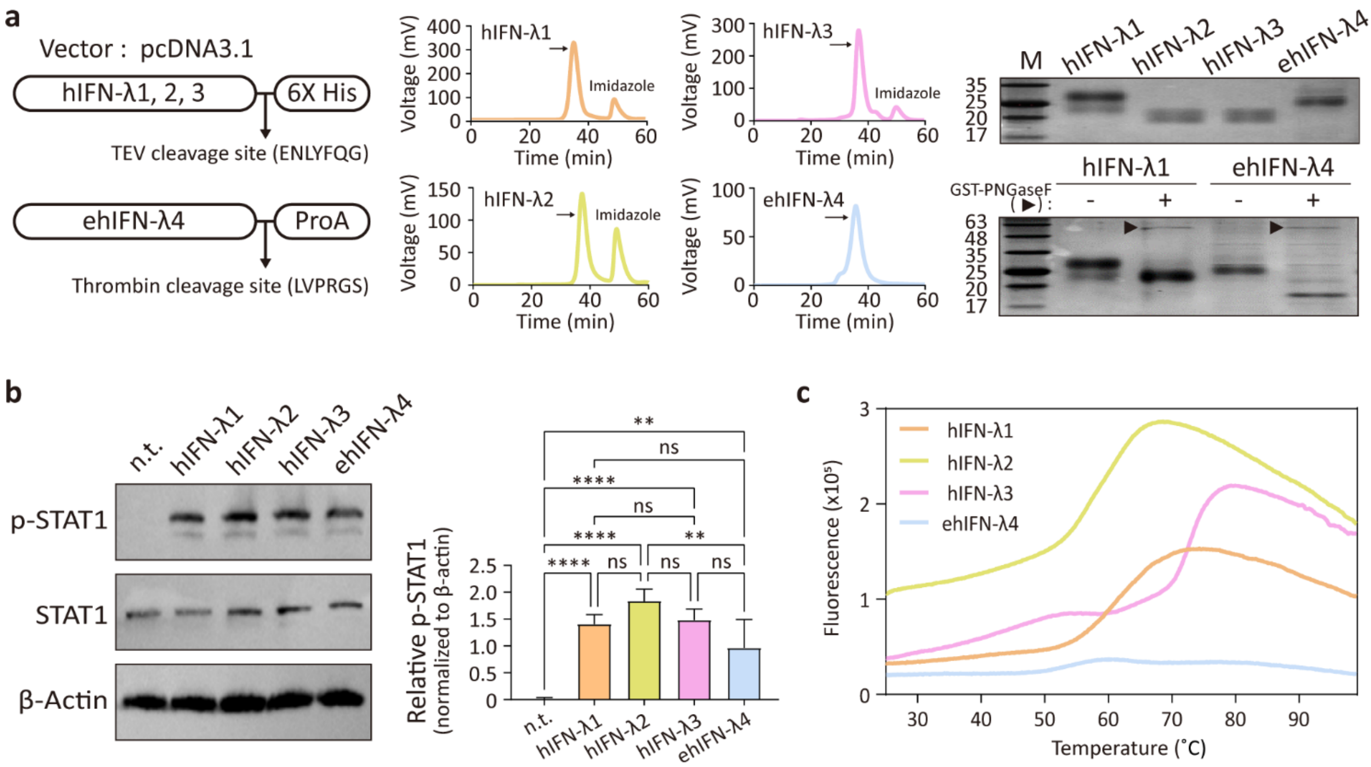

Figure S1. Comparative characterization of recombinant human IFN-λs for development of intranasal antiviral biologic

a Schematic diagram of expression constructs for recombinant hIFN-λ1, λ2, λ3 (with TEV-cleavable 6×His tag) and glycoengineered hIFN-λ4 (ehIFN-λ4, with thrombin-cleavable Protein A tag) (left). Representative chromatograms from size-exclusion chromatography (SEC) of purified hIFN-λs (middle). SDS-PAGE analysis of purified hIFN-λs and deglycosylated proteins treated with PNGaseF (ehIFN-λ4[29] is glyco-engineered hIFN-λ4) (right). M, molecular weight marker. **b** Immunoblot analysis of intracellular STAT1 signaling in HNEpCs treated with 100 ng/mL of each hIFN-λ for 1 h. Levels of phosphorylated STAT1 (p-STAT1), total STAT1 (t-STAT1), and β-actin were quantified by densitometry. Relative p-STAT1 is expressed as the ratio of p-STAT1 to t-STAT1, normalized to β-actin (n = 4). **c** Melting curve of hIFN-λ1, λ2, λ3, and ehIFN-λ4 measured by thermal shift assay. Each sample (12.5 µg) was incubated with Protein Thermal Shift™ dye, and fluorescence was monitored during gradual heating to determine melting temperatures (Tm). All data represent mean ± SD from independent experiments. Statistical significance was assessed using one-way ANOVA followed by Tukey's multiple comparisons test (**P < 0.01, ***P < 0.001, **P < 0.0001; ns, not significant).

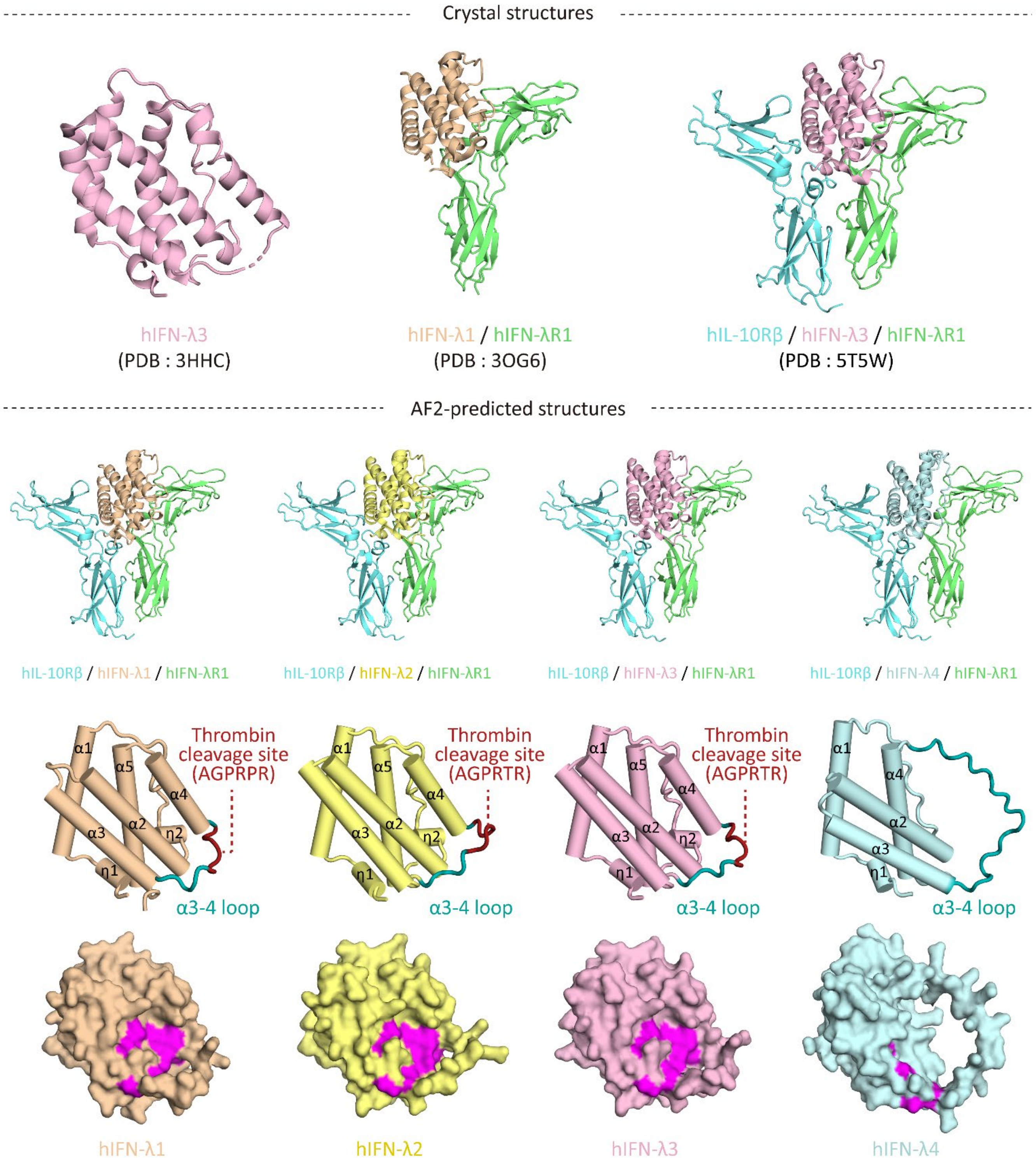

Figure S2. Structural comparison of human IFN-λs in apo form and receptor-bound complexes (hIL-10Rβ/hIFN-λs/hIFN-λR1)

Crystal structures of hIFN-λ3 alone (PDB: 3HHC) [49], the hIFN-λ1/hIFN-λR1 complex (PDB: 3OG6) [62], and the ternary complex of hIL-10Rβ/hIFN-λ3/hIFN-λR1 (PDB: 5T5W) [50] are shown (top). AlphaFold2-predicted structures of hIFN-λ1–4 in complex with their receptors (hIL-10Rβ and hIFN-λR1) are shown (bottom). Secondary structural elements, including α-helices and 3_{10} helices (η), and the thrombin cleavage site (red) are annotated in cartoon representation. The exposed hydrophobic patch adjacent to the α3–4 loop is highlighted in magenta in the surface view.

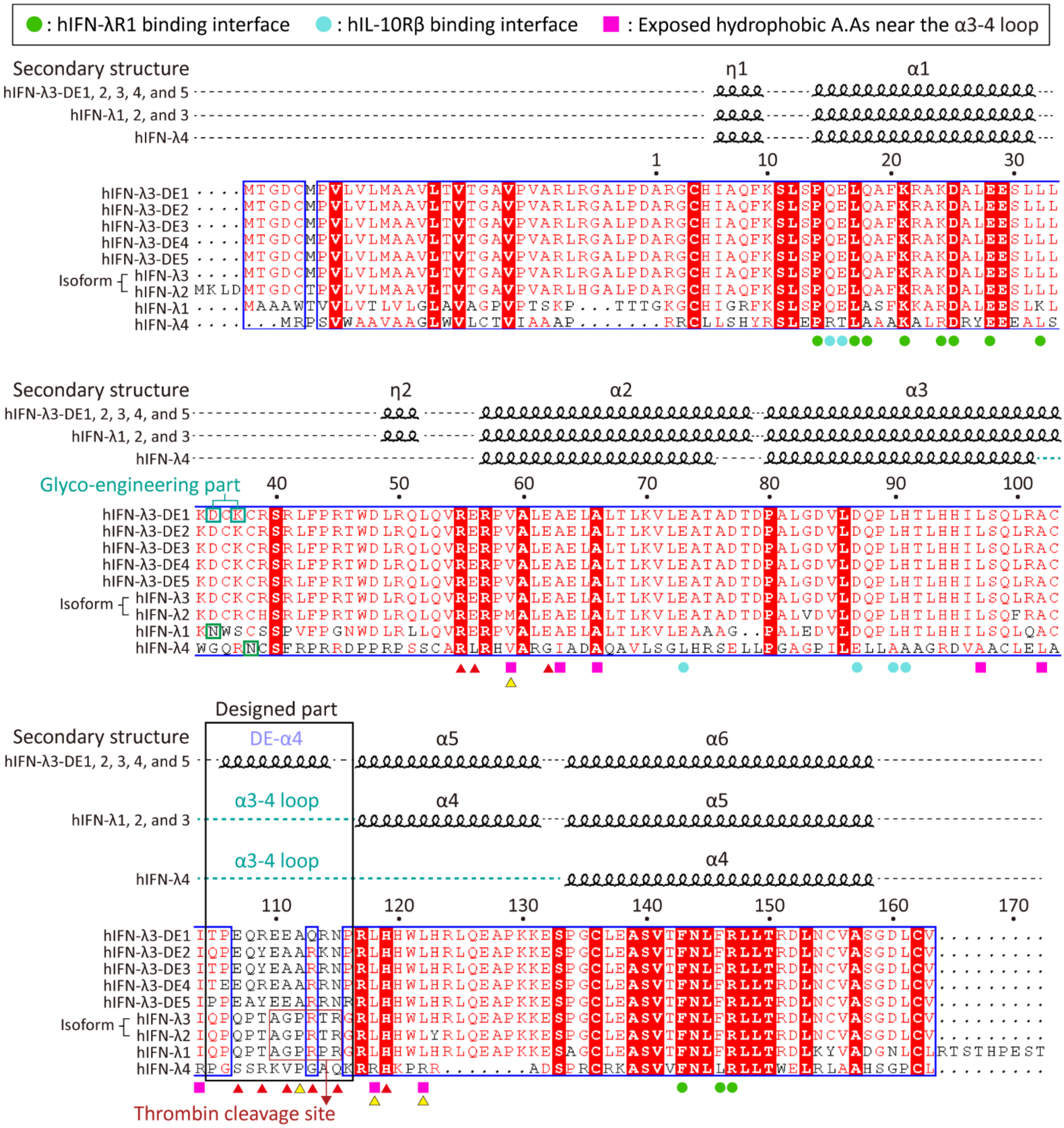

Figure S3. Sequence alignments of five hIFN-λ3 designs and natural hIFN-λs

Multiple sequence alignment of five designed hIFN-λ3 variants (DE1–DE5) with human IFN-λ1 (UniProt: Q8IU54), IFN-λ2 (Q8IZJ0), IFN-λ3 (Q8IZI9), and IFN-λ4 (K9M1U5). Secondary structural elements were assigned based on the crystal structures of hIFN-λ1 (PDB: 3OG6)[62] and hIFN-λ3 (PDB: 3HHC)[49], and AlphaFold2-predicted structures of hIFN-λ2 and hIFN-λ4. The sequence alignment was generated using ESPript (http://espript.ibcp.fr). Strictly conserved residues are shown in red with white lettering, and residues with >70% similarity are boxed in blue with red characters. α-helices and 3_{10}-helices (η) are indicated above the sequences. Functionally relevant features are annotated as follows: green boxes indicate native N-glycosylation sites in hIFN-λ1 and hIFN-λ4, or the glyco-engineered site in G-hIFN-λ3-DE1; magenta squares mark exposed hydrophobic residues near the α3–4 loop; red text denotes the thrombin cleavage sequence. Red and yellow triangles represent electrostatic and hydrophobic interactions, respectively, formed between the DE-α4 helix and adjacent α2 or α5 helices in the AF2-predicted model of hIFN-λ3-DE1. Residue numbering is based on the hIFN-λ3 crystal structure (Gad et al., 2009)[49].

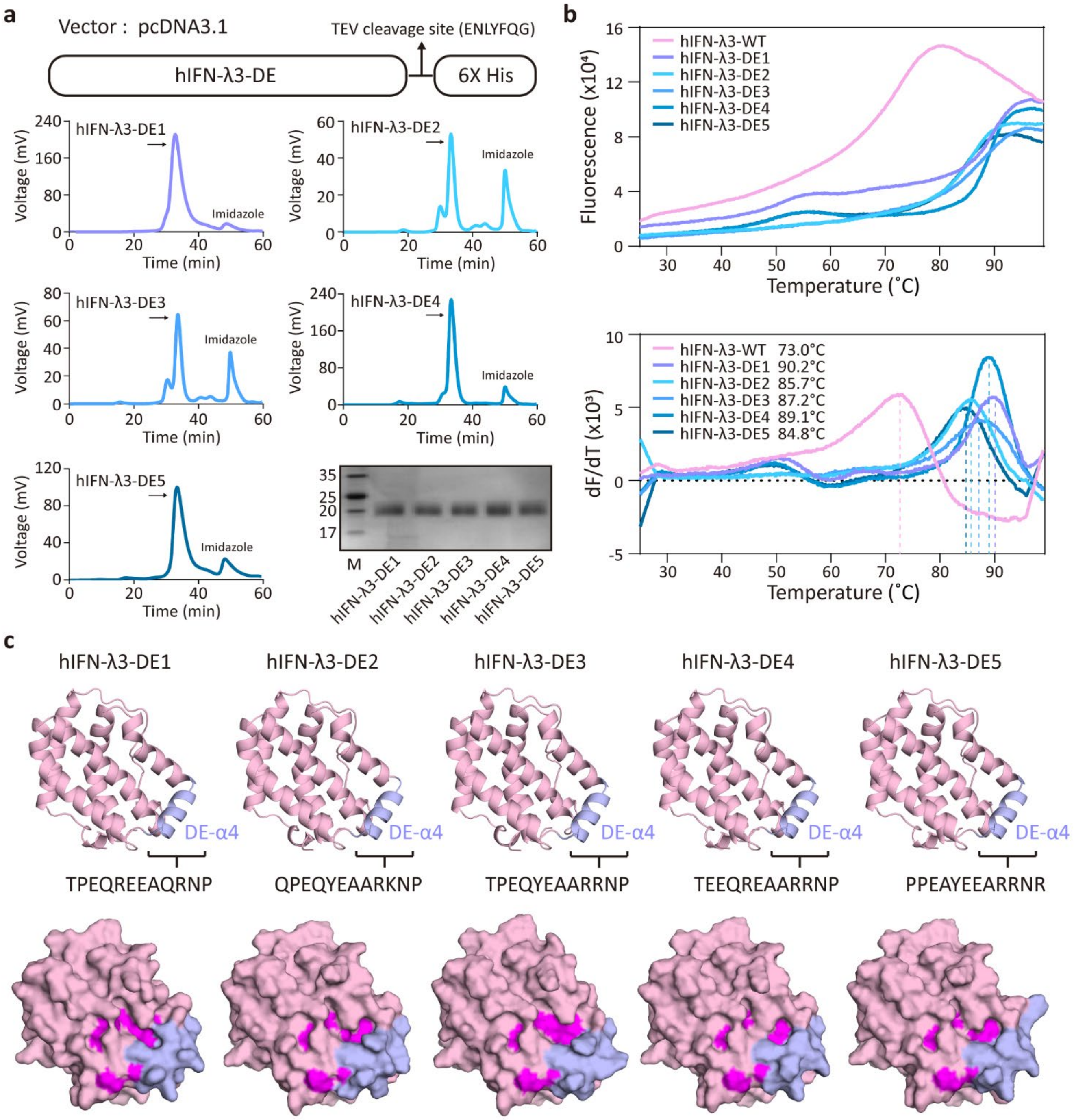

Figure S4. Purification, thermal stability, and structural features of five hIFN-λ3 design variants

a Schematic of the expression construct used to generate five hIFN-λ3 variants (DE1–DE5) containing a C-terminal 6X-His tag and TEV cleavage site (top). Representative chromatograms from size-exclusion chromatography (SEC) are shown for each variant, and purified proteins were analyzed by SDS-PAGE followed by Coomassie blue staining (bottom). **b** Thermal stability of hIFN-λ3-WT and the five hIFN-λ3-DE variants assessed by thermal shift assay. Each reaction contained 12.5 µg of protein and 2.5 µL of diluted Protein Thermal Shift™ Dye. Melting curve (top) and their first derivatives (bottom) are shown. Melting temperatures (Tm), determined as the temperature at which dF/dT peaked, are indicated. **c** AlphaFold2-predicted structural models of hIFN-λ3 design variants (DE1–DE5). Cartoon and surface representations are shown for each variant, highlighting the engineered DE-α4 helix (purple) and the exposed hydrophobic patch (magenta) in each variant.

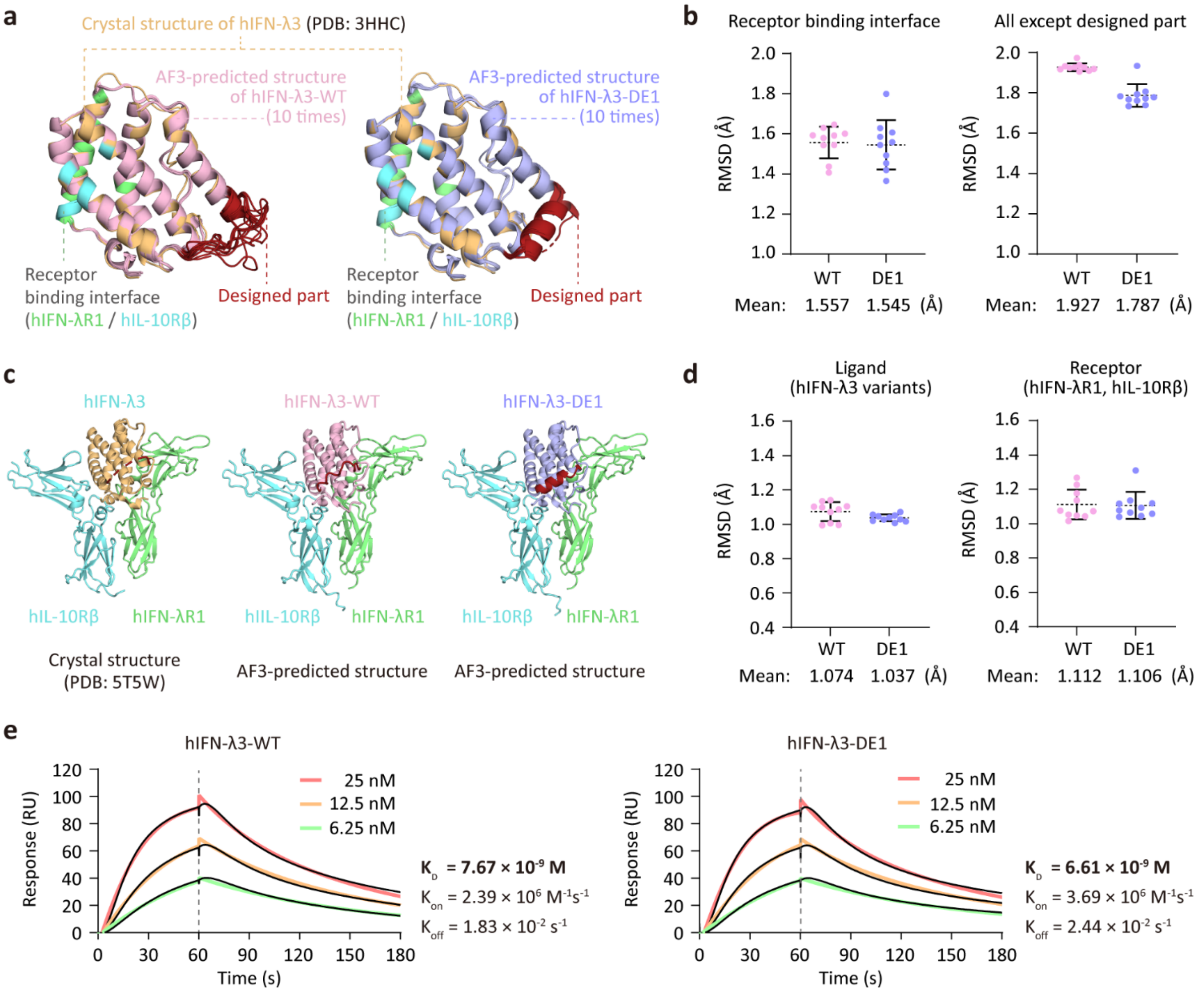

Figure S5. Binding affinity and structure comparison of wild-type and designed hIFN-λ3 variants

a Structural alignment of AlphaFold3 [48]-predicted models of hIFN-λ3-WT (pink) and hIFN-λ3-DE1 (light blue) with the crystal structure of wild-type hIFN-λ3 (light orange, PDB: 3HHC) [49]. **b** Local RMSD values for the receptor-binding interface and non-designed regions (all regions except the designed part) of hIFN-λ3-WT and hIFN-λ3-DE1 compared to the crystal structure. **c** The crystal structure of the wild-type hIFN-λ3-receptor complex (left; PDB: 5T5W) [50] and AlphaFold3-predicted ternary complex structures (hIFN-λ3 variant–hIFN-λR1–hIL-10Rβ). **d** Average RMSD values for the ligand (hIFN-λ3-WT or hIFN-λ3-DE1) and receptor components (hIFN-λR1 and hIL-10Rβ) from predicted complexes aligned to the crystal structure. **e** Binding kinetics of hIFN-λ-WT (left) and hIFN-λ3-DE1 (right) to the receptor hIFN-λR1 were determined by surface plasmon resonance (SPR). The equilibrium dissociation constant (KD, M) was calculated as the ratio of the dissociation rate constant (koff) to the association rate constant (kon). Kinetic parameters were obtained by global fitting to a 1:1 binding model using the Biacore Insight Evaluation Software.

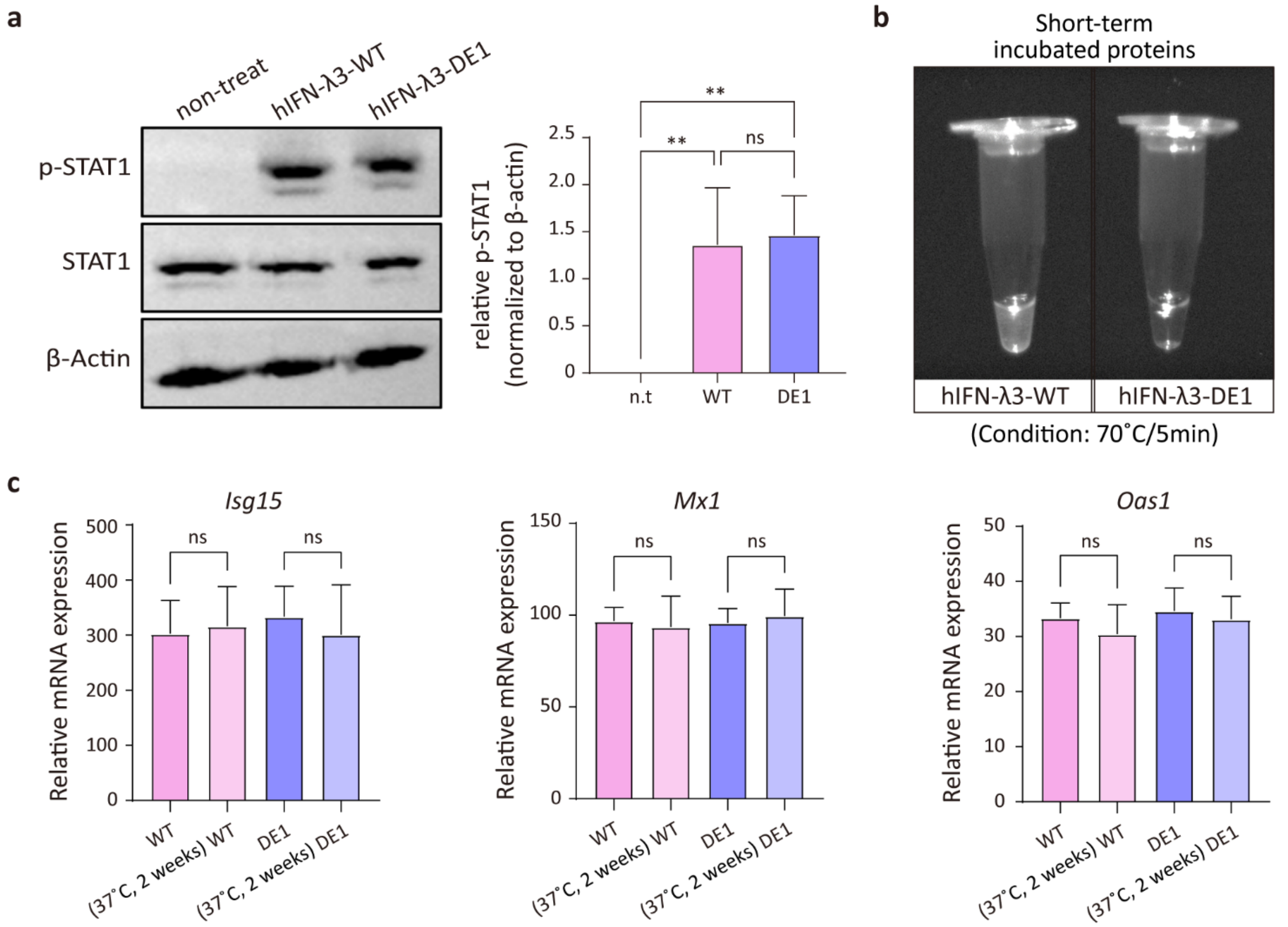

Figure S6. hIFN-λ3-DE1 exhibits enhanced thermal stability while retaining biological activity after prolonged incubation at 37°C

a Immunoblot analysis of STAT1 phosphorylation in HNEpCs stimulated with recombinant hIFN-λ3-WT or hIFN-λ3-DE1 (100 ng/mL, 1 h). Levels of phosphorylated STAT1 (p-STAT1), total STAT1 (t-STAT1), and β-actin were quantified by densitometry. Relative p-STAT1 is expressed as the ratio of p-STAT1 to t-STAT1, normalized to β-actin. (*n* = 4). **b** hIFN-λ3-WT exhibited visible aggregation and precipitation after a 5 min incubation at 70°C, whereas hIFN-λ3-DE1 remained soluble. **c** Relative mRNA expression of representative ISGs (*Isg15, Mx1, and Oas1*) in HNEpCs following 12 h treatment with hIFN-λ3-WT or hIFN-λ3-DE1 (100 ng/mL), with or without long-term incubation at 37°C for 2 weeks. mRNA levels were analyzed by RT-qPCR (*n* = 3), normalized to *18S rRNA*, and expressed relative to non-treated controls. All data represent mean ± SD from independent experiments. Statistical analysis was performed by one-way ANOVA test followed by Tukey's or Sidak's multiple comparisons test (0.001<***P*<0.01 vs. control and ns is not significant). n.t., non-treat; WT, hIFN-λ3-WT; DE1, hIFN-λ3-DE1.

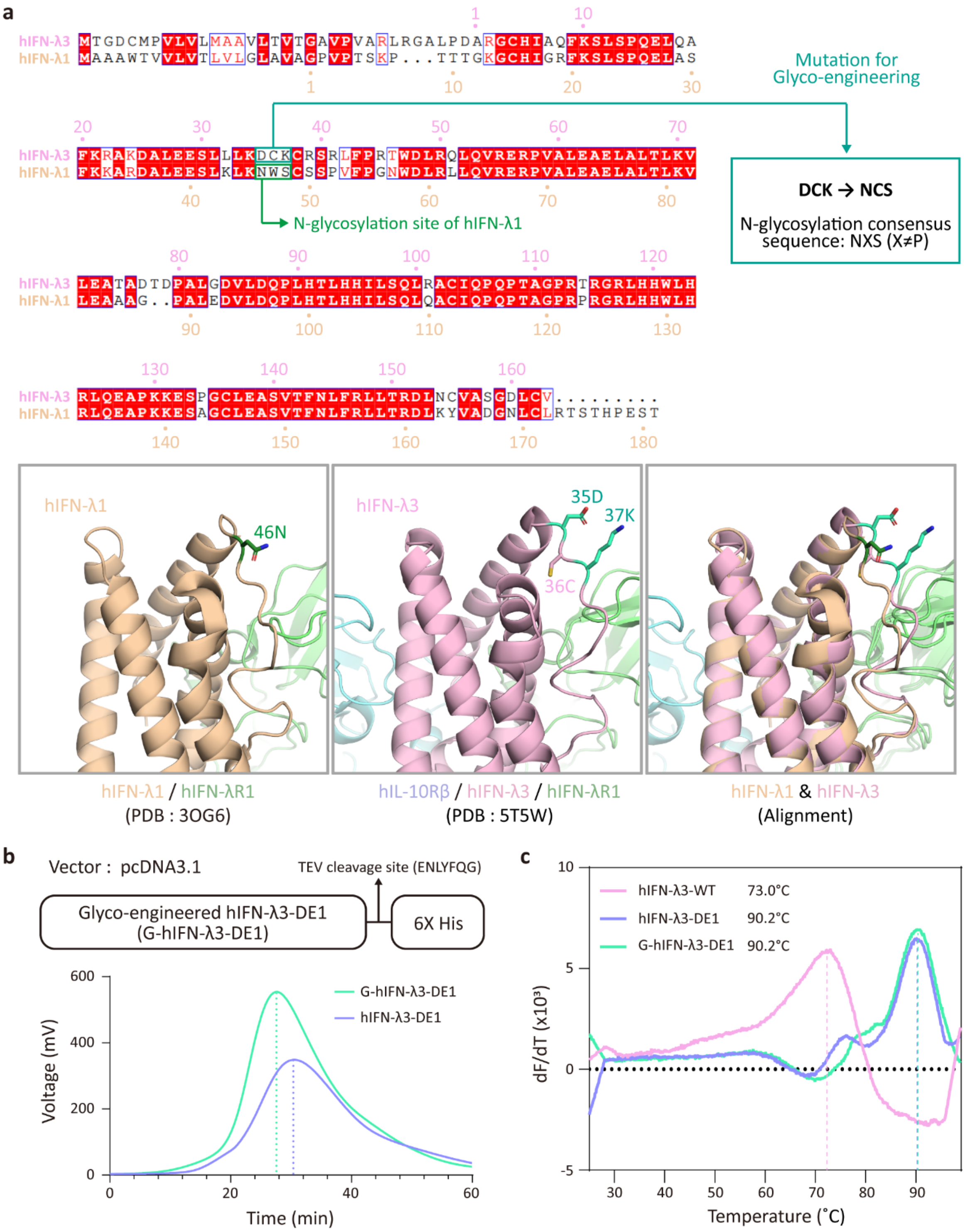

Figure S7. Structure-guided glyco-engineering of hIFN-λ3-DE1 and assessment of thermal stability

a Amino acid sequence alignment of human IFN-λ1 (UniProt: Q8IU54) and IFN-λ3 (UniProt: Q8IZI9). The sequence alignment was generated using ESPript (http://espript.ibcp.fr). Strictly conserved residues are shown in red with white lettering, and residues with >70% similarity are boxed in blue with red characters. α-helices and 3_{10}-helices (η) are indicated above the sequences. Residue numbering follows the crystal structures of hIFN-λ1 (PDB: 3OG6)[62] and hIFN-λ3 (PDB: 3HHC)[49]. The native N-glycosylation site of hIFN-λ1 and the glyco-engineered site in hIFN-λ3-DE1 (DCK → NCS) are highlighted with green boxes (top) and shown as sticks in the cartoon structure (bottom). Crystal structures of hIFN-λ1/hIFN-λR1 and hIFN-λ3/hIL-10Rβ/hIFN-λR1, and their structural alignment are shown to visualize glycosylation site locations. **b** Schematic of the expression construct for glyco-engineered hIFN-λ3-DE1 (G-hIFN-λ3-DE1) (top) and representative chromatograms from size-exclusion chromatography (SEC) for hIFN-λ3-DE1 and G-hIFN-λ3-DE1 are shown for comparison (bottom). **c** Thermal stability of G-hIFN-λ3-DE1 assessed by thermal shift assay. Each reaction contained 12.5 µg of protein and 2.5 µL of diluted Protein Thermal Shift™ Dye. The first derivatives (dF/dT, bottom) are shown. Melting temperatures (Tm), determined as the temperature at which dF/dT peaked, are indicated.

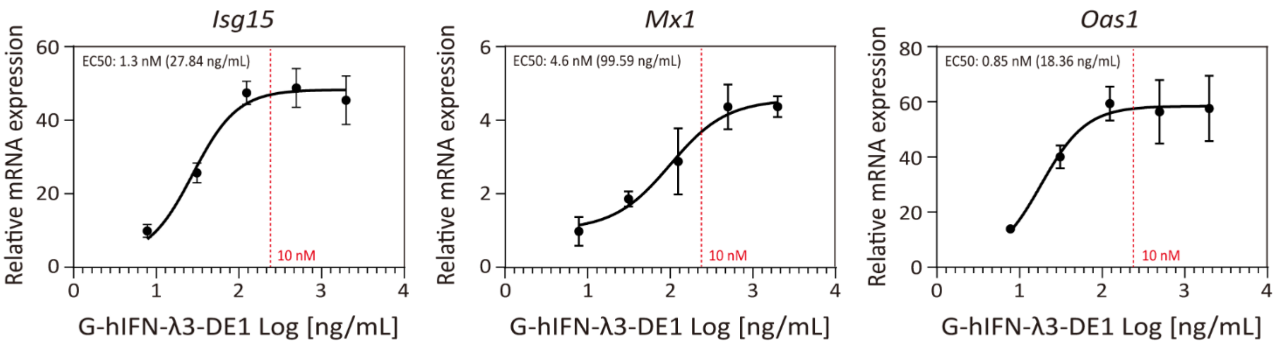

Figure S8. Dose-dependent induction of ISGs in Vero E6 cells treated with G-hIFN-λ3-DE1

Vero E6 cells were treated with increasing concentrations (7.8-2000 ng/mL; 4-fold serial dilution) of G-hIFN-λ3-DE1 for 24 h. Relative mRNA levels of representative ISGs (*Isg15*, *Mx1*, and *Oas1*) were analyzed by RT-qPCR, normalized to *Gapdh*, and expressed relative to non-treated controls. All data represent mean ± SEM from independent experiments (*n* = 3). Curves are four-parameter logistic fits (GraphPad Prism 8.0), EC_{50} values are indicated in each panel, and the red dashed line indicates the concentration corresponding to 10 nM G-hIFN-λ3-DE1.

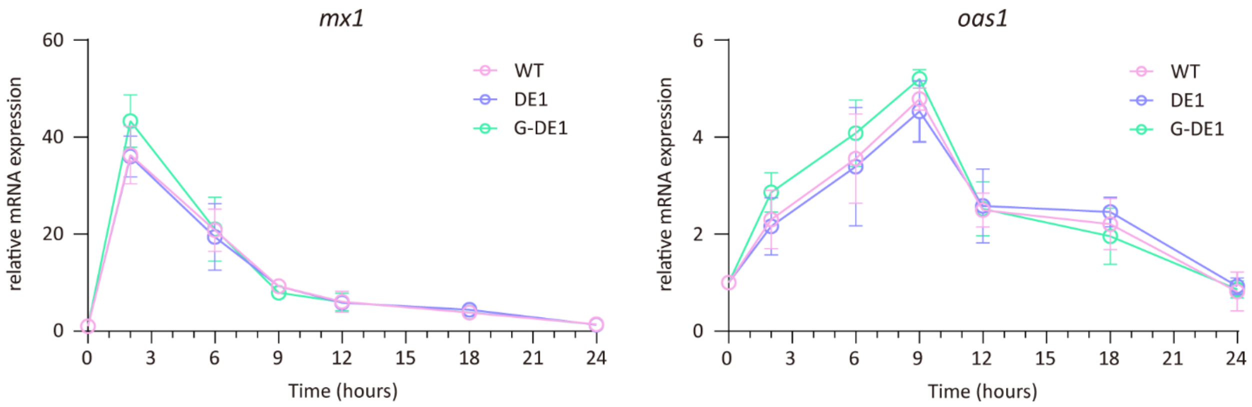

Figure S9. Temporal induction of *mx1* and *oas1* in mouse nasal turbinates after intranasal administration of hIFN-λ3 variants
Time-course analysis of *mx1* and *oas1* expression in mouse nasal turbinates following intranasal administration of recombinant hIFN-λ3-WT, hIFN-λ3-DE1, or G-hIFN-λ3-DE1 (5 μg in 10 μL PBS; 0.5 mg/mL). Nasal tissues were collected at the indicated time points (0–24 h). Relative mRNA levels were quantified by RT-qPCR (n = 3), normalized to mouse *gapdh*, and expressed relative to non-treated controls. All data represent mean± SD from independent experiments. WT, hIFN-λ3-WT; DE1, hIFN-λ3-DE1; G-DE1, G-hIFN-λ3-DE1.

Table S1: Primer sequences for RT-qPCR

Gene name	Primer	Primer sequence (5' → 3')
18s (human)	Forward	GTTGGTGGAGCGATTTGTCT
	Reverse	GGCCTCACTAAACCATCCAA
Isg15 (human)	Forward	TCCTGCTGGTGGTGGACAA
	Reverse	TTGTTATTCCTCACCAGGATGCT
Mx1 (human)	Forward	CCGTGACGGATATGGTCCGGC
	Reverse	CTGGAAGTGGAGGCGGATCAGC
Oas1 (human)	Forward	ATAAAAGCAAACAGGTCTGG
	Reverse	TCTGGCAAGAGATAGTCTTC
gapdh (mouse)	Forward	TCCATGACAACTTTGGCATT
	Reverse	GTTGCTGTTGAAGTCGCAGG
isg15 (mouse)	Forward	CATCCTGGTGAGGAACGAAAGG
	Reverse	CTCAGCCAGAACTGGTCTTCGT
mx1 (mouse)	Forward	GAGGTGGAGTTTGATGTGCTGC
	Reverse	GTGAAGCAGGTAGAGAACTCGC
oas1 (mouse)	Forward	TGGACATTGCTACCACAGAGGC
	Reverse	TTGCCTTCAGCACCTCTGTCCA
Gapdh (green monkey)	Forward	CTGGGCTACACTGAGCACC
	Reverse	AAGTGGTCGTTGAGGGCAATG
Isg15 (green monkey)	Forward	TGGACAGATGCGATGAACCTC
	Reverse	GTCAGCTGTACCTCGTAGGTG
Mx1 (green monkey)	Forward	CCGTGACGGATATGGTCCGGC
	Reverse	CTGGAAGTGGAGGCGGATCAGC
Oas1 (green monkey)	Forward	ATAAAAGTGAACAGGTCTGG
	Reverse	TCTGGCAAGAGATAGTCTTC

Table S2: Amino acid sequence of recombinant hIFN-λs and designed hIFN-λ3s

Protein name	Amino acid sequence
hIFN-λ1	MAAAWTVVLVTLVLGLAVAGPVPTSKPTTTGKGCHIGRFKSLSPQELASFKKARDALEESLKLKNWSCSSPVFPG<br>NWDLRLLQVRERPVALEAELALTLKVLEAAAGPALEDVLDQPLHTLHHILSQLQACIQPQPTAGPRPRGRLHHW<br>LHRLQEAPKKESAGCLEASVTFNLFRLLTRDLKYVADGNLCLRTSTHPEST
hIFN-λ2	MKLDMTGDCTPVLVLMAAVLTVTGAVPVARLHGALPDARGCHIAQFKSLSPQELQAFKRAKDALEESLLLKDC<br>RCHSRLFPRTWDLRQLQVRERPMALEAELALTLKVLEATADTDPALVDVLDQPLHTLHHILSQFRACIQPQPTA<br>GPRTRGRLHHWLYRLQEAPKKESPGCLEASVTFNLFRLLTRDLNCVASGDLCV
hIFN-λ3	MTGDCMPVLVLMAAVLTVTGAVPVARLRGALPDARGCHIAQFKSLSPQELQAFKRAKDALEESLLLK**DCK**CRS<br>RLFPRTWDLRQLQVRERPVALEAELALTLKVLEATADTDPALGDVLDQPLHTLHHILSQLRACI**QPQPTAGPRTR**<br>**G**RLHHWLHRLQEAPKKESPGCLEASVTFNLFRLLTRDLNCVASGDLCV
ehIFN-λ4	MRPSVWAAVAAGLWVLCTVIAAAPRRCNLSHYRSLEPRTLAAAKALRDRYEEEALSWGQRNCSFRPRRDPPR<br>NSSCARLRHVARGIADAQAVLSGLHRSELLPGAGPILELLAAAGRDVAACLELARPGSSRKVPGAQKRRHKPRR<br>ADSPRCRKASVVFNLLRLLTWELRLAAHSGPCL
hIFN-λ3-DE1	MTGDCMPVLVLMAAVLTVTGAVPVARLRGALPDARGCHIAQFKSLSPQELQAFKRAKDALEESLLLKDCKCRS<br>RLFPRTWDLRQLQVRERPVALEAELALTLKVLEATADTDPALGDVLDQPLHTLHHILSQLRACI**TPEQREEAQRN**<br>**P**RLHHWLHRLQEAPKKESPGCLEASVTFNLFRLLTRDLNCVASGDLCV
hIFN-λ3-DE2	MTGDCMPVLVLMAAVLTVTGAVPVARLRGALPDARGCHIAQFKSLSPQELQAFKRAKDALEESLLLKDCKCRS<br>RLFPRTWDLRQLQVRERPVALEAELALTLKVLEATADTDPALGDVLDQPLHTLHHILSQLRACI**QPEQYEAARKN**<br>**P**RLHHWLHRLQEAPKKESPGCLEASVTFNLFRLLTRDLNCVASGDLCV
hIFN-λ3-DE3	MTGDCMPVLVLMAAVLTVTGAVPVARLRGALPDARGCHIAQFKSLSPQELQAFKRAKDALEESLLLKDCKCRS<br>RLFPRTWDLRQLQVRERPVALEAELALTLKVLEATADTDPALGDVLDQPLHTLHHILSQLRACI**TPEQYEAARRN**<br>**P**RLHHWLHRLQEAPKKESPGCLEASVTFNLFRLLTRDLNCVASGDLCV
hIFN-λ3-DE4	MTGDCMPVLVLMAAVLTVTGAVPVARLRGALPDARGCHIAQFKSLSPQELQAFKRAKDALEESLLLKDCKCRS<br>RLFPRTWDLRQLQVRERPVALEAELALTLKVLEATADTDPALGDVLDQPLHTLHHILSQLRACI**TEEQREAARRN**<br>**P**RLHHWLHRLQEAPKKESPGCLEASVTFNLFRLLTRDLNCVASGDLCV
hIFN-λ3-DE5	MTGDCMPVLVLMAAVLTVTGAVPVARLRGALPDARGCHIAQFKSLSPQELQAFKRAKDALEESLLLKDCKCRS<br>RLFPRTWDLRQLQVRERPVALEAELALTLKVLEATADTDPALGDVLDQPLHTLHHILSQLRACI**PPEAYEEARRN**<br>**R**RLHHWLHRLQEAPKKESPGCLEASVTFNLFRLLTRDLNCVASGDLCV
G-hIFN-λ3-DE1	MTGDCMPVLVLMAAVLTVTGAVPVARLRGALPDARGCHIAQFKSLSPQELQAFKRAKDALEESLLLK**NCS**CRS<br>RLFPRTWDLRQLQVRERPVALEAELALTLKVLEATADTDPALGDVLDQPLHTLHHILSQLRACI**TPEQREEAQRN**<br>**P**RLHHWLHRLQEAPKKESPGCLEASVTFNLFRLLTRDLNCVASGDLCV

***Designed part *Glyco-engineering part**

Table S3: DNA sequence of recombinant hIFN-λs and designed hIFN-λ3s

Gene name	DNA sequence
hIFN-λ1	ATGGCCGCAGCATGGACAGTAGTTTTAGTAACCCTGGTCCTCGGACTGGCTGTAGCCGGGCCGGTTCCTAC<br>CTCTAAACCTACTACTACGGGTAAAGGTTGTCATATAGGTCGGTTTAAGAGTCTTTCCCCTCAAGAATTGGC<br>TTCCTTTAAGAAAGCGCGTGATGCTCTTGAGGAAAGCCTGAAATTAAAGAATTGGTCATGTAGTTCACCAG<br>TATTTCCTGGCAACTGGGATTTGCGCTTGCTGCAAGTAAGAGAAAGGCCAGTTGCACTTGAAGCCGAACTC<br>GCACTTACATTGAAAGTGCTTGAAGCCGCCGCCGGACCTGCTCTCGAAGATGTACTGGATCAACCATTGCA<br>TACTCTCCATCATATTCTGAGTCAATTGCAAGCATGCATACAACCACAACCGACCGCTGGCCCTCGTCCTA<br>GAGGAAGACTGCATCATTGGTTACATAGACTGCAAGAAGCTCCAAAGAAAGAAAGCGCCGGATGTCTCGA<br>AGCCAGCGTGACGTT TAATCTGTTTCGGTTGCTGACTAGAGATTTGAAGTACGTTGCGGACGGCAATCTTT<br>GTCTCCGCACCTCTACTCATCCCGAATCTACG
hIFN-λ2	ATGAAACTTGATATGACTGGCGATTGTACCCCTGTCCTGGTCTTAATGGCTGCCGTCCTCACTGTTACAGGC<br>GCCGTACCAGTTGCGCGGCTGCATGGCGCCTTGCCAGACGCTCGCGGATGTCATATCGCTCAATTTAAATC<br>TCTCTCCCCACAAGAACTTCAAGCGTTCAAACGCGCAAAGGACGCGCTCGAAGAAAGCCTGTTGCTCAAA<br>GATTGTCGGTGTCATAGCAGACTGTTTCCACGCACTTGGGATCTCCGCCAACTTCAAGTCCGCGAAAGACC<br>TATGGCCCTCGAAGCGGAACTTGCGCTCACCTTGAAAGTGCTTGAAGCTACTGCCGATACCGATCCTGCTC<br>TTGTAGATGTACTGGATCAACCTCTGCATACATTGCATCACATACTTTCTCAATTTAGAGCGTGCATACAAC<br>CCCAACCAACAGCTGGTCCGCGGACACGAGGGAGATTGCATCACTGGTTATATCGACTTCAAGAAGCTCC<br>TAAGAAAGAAAGCCCCGGTTGTCTGGAAGCATCAGTAACGTTTAATCTGTTTAGACTGTTGACACGTGATC<br>TCAACTGCGTCGCGTCCGGCGATTTGTGCGTG
hIFN-λ3	ATGACTGGTGATTGTATGCCCGTGCTCGTTCTGATGGCTGCGGTTTTAACAGTCACAGGTGCGGTGCCAGT<br>GGCACGACTGCGTGGCGCCTTGCCCGACGCTCGGGGTTGTCATATCGCTCAATTTAAAAGTCTTTCACCCC<br>AAGAACTTCAAGCGTTCAAAAGAGCGAAGGACGCGCTTGAGGAAAGTTTATTATTGAAA**GATTGTAAA**TGT<br>CGATCAAGACTGTTTCCGCGCACGTGGGATCTCCGTCAACTCC AAGTCCGGGAAAGACCAGTAGCACTGG<br>AAGCAGAACTCGCTTTGACCCTCAAAGTGTTGGAAGCTACTGCCGATACCGATCCCGCTTTAGGCGACGTG<br>CTTGATCAACCACTGCATACATTGCATCACATTTTGAGTCAATTAAGAGCATGCATT**CAACCCCAACCAACC**<br>**GCCGGTCCAAGAACAAGAGGG**AGATTGCATCACTGGCTCCATAGGTTGCAAGAAGCTCCGAAGAAAGAA<br>TCACCAGGATGTCTTGAAGCGTCCGTTACGTTTAATCTGTTTCGGCTGCTTACCAGGGATCTTAACTGCGTG<br>GCTAGTGGAGATCTCTGCGTT
ehIFN-λ4	ATGAGACCTTCCGTCTGGGCCGCCGTGGCCGCAGGACTGTGGGTCCTGTGCACCGTGATCGCCGCAGCCC<br>CTAGAAGATGCAATCTCTCCCACTACCGCAGCCTGGAGCCCAGAACACTGGCCGCTGCCAAGGCCCTGAG<br>GGACAGATATGAGGAAGAAGCCCTGAGCTGGGGGCAGCGGAACTGCTCCTTCCGGCCCAGGAGGGACCC<br>TCCAAGGAATAGCTCCTGCGCTAGGCTCAGGCACGTGGCTAGGGGAATCGCCGACGCTCAGGCTGTGCTT<br>AGCGGCCTTCACAGGTCCGAGCTGCTCCCTGGCGCTGGCCCAATTCTGGAGCTGCTGGCCGCAGCAGGGA<br>GGGATGTGGCCGCCTGTCTTGAGTTGGCCAGGCCAGGCTCTAGTCGGAAGGTCCCCGGGGCCCAAAAGC<br>GGCGCCATAAACCCCGGCGGGCCGATTCACCCCGGTGTCGGAAGGCCTCTGTGGTCTTTAATCTCCTCCG<br>GCTCCTGACTTGGGAGTTGCGCTTGGCCGCCCACAGCGGGCCCTGCTTG
hIFN-λ3-DE1	ATGACTGGTGATTGTATGCCCGTGCTCGTTCTGATGGCTGCGGTTTTAACAGTCACAGGTGCGGTGCCAGT<br>GGCACGACTGCGTGGCGCCTTGCCCGACGCTCGGGGTTGTCATATCGCTCAATTTAAAAGTCTTTCACCCC<br>AAGAACTTCAAGCGTTCAAAAGAGCGAAGGACGCGCTTGAGGAAAGTTTATTATTGAAAGATTGTAAATGT<br>CGATCAAGACTGTTTCCGCGCACGTGGGATCTCCGTCAACTCCAAGTCCGGGAAAGACCAGTAGCACTGG<br>AAGCAGAACTCGCTTTGACCCTCAAAGTGTTGGAAGCTACTGCCGATACCGATCCCGCTTTAGGCGACGTG<br>CTTGATCAACCACTGCATACATTGCATCACATTTTGAGTCAATTAAGAGCATGCATT**ACACCAGAGCAGAG**<br>**AGAGGAAGCCCAGAGAAATCCT**AGATTGCATCACTGGCTCCATAGGTTGCAAGAAGCTCCGAAGAAAGA<br>ATCACCAGGATGTCTTGAAGCGTCCGTTACGTTTAATCTGTTTCGGCTGCTTACCAGGGATCTTAACTGCGT<br>GGCTAGTGGAGATCTCTGCGTT
hIFN-λ3-DE2	ATGACTGGTGATTGTATGCCCGTGCTCGTTCTGATGGCTGCGGTTTTAACAGTCACAGGTGCGGTGCCAGT<br>GGCACGACTGCGTGGCGCCTTGCCCGACGCTCGGGGTTGTCATATCGCTCAATTTAAAAGTCTTTCACCCC<br>AAGAACTTCAAGCGTTCAAAAGAGCGAAGGACGCGCTTGAGGAAAGTTTATTATTGAAAGATTGTAAATGT<br>CGATCAAGACTGTTTCCGCGCACGTGGGATCTCCGTCAACTCC AAGTCCGGGAAAGACCAGTAGCACTGG<br>AAGCAGAACTCGCTTTGACCCTCAAAGTGTTGGAAGCTACTGCCGATACCGATCCCGCTTTAGGCGACGTG<br>CTTGATCAACCACTGCATACATTGCATCACATTTTGAGTCAATTAAGAGCATGCATT**CAGCCCGAGCAGTAT**<br>**GAAGCCGCTAGAAAGAATCCT**AGATTGCATCACTGGCTCCATAGGTTGCAAGAAGCTCCGAAGAAAGAAT<br>CACCAGGATGTCTTGAAGCGTCCGTTACGTTTAATCTGTTTCGGCTGCTTACCAGGGATCTTAACTGCGTGG<br>CTAGTGGAGATCTCTGCGTT
hIFN-λ3-DE3	ATGACTGGTGATTGTATGCCCGTGCTCGTTCTGATGGCTGCGGTTTTAACAGTCACAGGTGCGGTGCCAGT<br>GGCACGACTGCGTGGCGCCTTGCCCGACGCTCGGGGTTGTCATATCGCTCAATTTAAAAGTCTTTCACCCC<br>AAGAACTTCAAGCGTTCAAAAGAGCGAAGGACGCGCTTGAGGAAAGTTTATTATTGAAAGATTGTAAATGT<br>CGATCAAGACTGTTTCCGCGCACGTGGGATCTCCGTCAACTCC AAGTCCGGGAAAGACCAGTAGCACTGG<br>AAGCAGAACTCGCTTTGACCCTCAAAGTGTTGGAAGCTACTGCCGATACCGATCCCGCTTTAGGCGACGTG<br>CTTGATCAACCACTGCATACATTGCATCACATTTTGAGTCAATTAAGAGCATGCATT**ACCCCTGAGCAGTAC**<br>**GAAGCCGCTAGAAGAAATCCA**AGATTGCATCACTGGCTCCATAGGTTGCAAGAAGCTCCGAAGAAAGAAT<br>CACCAGGATGTCTTGAAGCGTCCGTTACGTTTAATCTGTTTCGGCTGCTTACCAGGGATCTTAACTGCGTGG<br>CTAGTGGAGATCTCTGCGTT
hIFN-λ3-DE4	ATGACTGGTGATTGTATGCCCGTGCTCGTTCTGATGGCTGCGGTTTTAACAGTCACAGGTGCGGTGCCAGT<br>GGCACGACTGCGTGGCGCCTTGCCCGACGCTCGGGGTTGTCATATCGCTCAATTTAAAAGTCTTTCACCCC<br>AAGAACTTCAAGCGTTCAAAAGAGCGAAGGACGCGCTTGAGGAAAGTTTATTATTGAAAGATTGTAAATGT<br>CGATCAAGACTGTTTCCGCGCACGTGGGATCTCCGTCAACTCC AAGTCCGGGAAAGACCAGTAGCACTGG<br>AAGCAGAACTCGCTTTGACCCTCAAAGTGTTGGAAGCTACTGCCGATACCGATCCCGCTTTAGGCGACGTG<br>CTTGATCAACCACTGCATACATTGCATCACATTTTGAGTCAATTAAGAGCATGCATT**ACCGAGGAACAGAG**<br>**AGAGGCTGCCAGAAGAAATCCA**AGATTGCATCACTGGCTCCATAGGTTGCAAGAAGCTCCGAAGAAAGA<br>ATCACCAGGATGTCTTGAAGCGTCCGTTACGTTTAATCTGTTTCGGCTGCTTACCAGGGATCTTAACTGCGT<br>GGCTAGTGGAGATCTCTGCGTT
hIFN-λ3-DE5	ATGACTGGTGATTGTATGCCCGTGCTCGTTCTGATGGCTGCGGTTTTAACAGTCACAGGTGCGGTGCCAGT<br>GGCACGACTGCGTGGCGCCTTGCCCGACGCTCGGGGTTGTCATATCGCTCAATTTAAAAGTCTTTCACCCC<br>AAGAACTTCAAGCGTTCAAAAGAGCGAAGGACGCGCTTGAGGAAAGTTTATTATTGAAAGATTGTAAATGT<br>CGATCAAGACTGTTTCCGCGCACGTGGGATCTCCGTCAACTCC AAGTCCGGGAAAGACCAGTAGCACTGG<br>AAGCAGAACTCGCTTTGACCCTCAAAGTGTTGGAAGCTACTGCCGATACCGATCCCGCTTTAGGCGACGTG<br>CTTGATCAACCACTGCATACATTGCATCACATTTTGAGTCAATTAAGAGCATGCATT**CCCCCTGAAGCCTAC**<br>**GAGGAGGCTAGAAGAAATAGA**AGATTGCATCACTGGCTCCATAGGTTGCAAGAAGCTCCGAAGAAAGAA<br>TCACCAGGATGTCTTGAAGCGTCCGTTACGTTTAATCTGTTTCGGCTGCTTACCAGGGATCTTAACTGCGTG<br>GCTAGTGGAGATCTCTGCGTT
G-hIFN-λ3-DE1	ATGACTGGTGATTGTATGCCCGTGCTCGTTCTGATGGCTGCGGTTTTAACAGTCACAGGTGCGGTGCCAGT<br>GGCACGACTGCGTGGCGCCTTGCCCGACGCTCGGGGTTGTCATATCGCTCAATTTAAAAGTCTTTCACCCC<br>AAGAACTTCAAGCGTTCAAAAGAGCGAAGGACGCGCTTGAGGAAAGTTTATTATTGAAA**AACTGTAGT**TGT<br>CGATCAAGACTGTTTCCGCGCACGTGGGATCTCCGTCAACTCCAAGTCCGGGAAAGACCAGTAGCACTGG<br>AAGCAGAACTCGCTTTGACCCTCAAAGTGTTGGAAGCTACTGCCGATACCGATCCCGCTTTAGGCGACGTG<br>CTTGATCAACCACTGCATACATTGCATCACATTTTGAGTCAATTAAGAGCATGCATT**ACACCAGAGCAGAG**<br>**AGAGGAAGCCCAGAGAAATCCT**AGATTGCATCACTGGCTCCATAGGTTGCAAGAAGCTCCGAAGAAAGA<br>ATCACCAGGATGTCTTGAAGCGTCCGTTACGTTTAATCTGTTTCGGCTGCTTACCAGGGATCTTAACTGCGT<br>GGCTAGTGGAGATCTCTGCGTT

DNA sequences were codon optimized for protein expression, ***Designed part,** ***Glyco-engineering part**
